# Streptothricin F is a bactericidal antibiotic effective against highly drug-resistant gram-negative bacteria that interacts with the 30S subunit of the 70S ribosome

**DOI:** 10.1371/journal.pbio.3002091

**Published:** 2023-05-16

**Authors:** Christopher E. Morgan, Yoon-Suk Kang, Alex B. Green, Kenneth P. Smith, Matthew G. Dowgiallo, Brandon C. Miller, Lucius Chiaraviglio, Katherine A. Truelson, Katelyn E. Zulauf, Shade Rodriguez, Anthony D. Kang, Roman Manetsch, Edward W. Yu, James E. Kirby

**Affiliations:** 1 Department of Pharmacology, Case Western Reserve University School of Medicine, Cleveland, Ohio, United States of America; 2 Department of Pathology, Beth Israel Deaconess Medical Center, Boston, Massachusetts, United States of America; 3 Harvard Medical School, Boston, Massachusetts, United States of America; 4 Department of Chemistry and Chemical Biology, Northeastern University, Boston, Massachusetts, United States of America; 5 Department of Pharmaceutical Sciences, Northeastern University, Boston, Massachusetts, United States of America; 6 Center for Drug Discovery, Northeastern University, Boston, Massachusetts, United States of America; Biological Research Centre, HUNGARY

## Abstract

The streptothricin natural product mixture (also known as nourseothricin) was discovered in the early 1940s, generating intense initial interest because of excellent gram-negative activity. Here, we establish the activity spectrum of nourseothricin and its main components, streptothricin F (S-F, 1 lysine) and streptothricin D (S-D, 3 lysines), purified to homogeneity, against highly drug-resistant, carbapenem-resistant Enterobacterales (CRE) and *Acinetobacter baumannii*. For CRE, the MIC_50_ and MIC_90_ for S-F and S-D were 2 and 4 μM, and 0.25 and 0.5 μM, respectively. S-F and nourseothricin showed rapid, bactericidal activity. S-F and S-D both showed approximately 40-fold greater selectivity for prokaryotic than eukaryotic ribosomes in in vitro translation assays. In vivo, delayed renal toxicity occurred at >10-fold higher doses of S-F compared with S-D. Substantial treatment effect of S-F in the murine thigh model was observed against the otherwise pandrug-resistant, NDM-1-expressing *Klebsiella pneumoniae* Nevada strain with minimal or no toxicity. Cryo-EM characterization of S-F bound to the *A*. *baumannii* 70S ribosome defines extensive hydrogen bonding of the S-F steptolidine moiety, as a guanine mimetic, to the 16S rRNA C1054 nucleobase (*Escherichia coli* numbering) in helix 34, and the carbamoylated gulosamine moiety of S-F with A1196, explaining the high-level resistance conferred by corresponding mutations at the residues identified in single *rrn* operon *E*. *coli*. Structural analysis suggests that S-F probes the A-decoding site, which potentially may account for its miscoding activity. Based on unique and promising activity, we suggest that the streptothricin scaffold deserves further preclinical exploration as a potential therapeutic for drug-resistant, gram-negative pathogens.

## Abstract 2

Streptothricins were discovered in the early 1940’s to have excellent gram-negative antibiotic activity, but how do they work? A combination of microbiological and structural biology studies reveals their mechanism of action, highlighting unique interactions with the 30S ribosomal subunit.

## Introduction

The rapid emergence of antimicrobial resistance presents a significant challenge for treatment of bacterial infections. Carbapenem-resistant Enterobacterales (CRE) and *Acinetobacter baumannii* are of particular concern. Almost all approved antimicrobials that can overcome the gram-negative permeability barrier are natural products or synthetic or semisynthetic derivatives of natural products. Small molecules commonly available in high-throughput screening libraries rarely share similar physicochemical properties associated with gram-negative penetrance and activity [1]. Therefore, high-throughput screening efforts to identify novel antimicrobials using synthetic chemical libraries with rare exceptions have been nonproductive [1]. As a result, there is a significant antimicrobial discovery void.

Moreover, there is little doubt that resistance will emerge to agents currently in the pipeline. We are therefore clearly in need of several new gram-negative agents that are unique in terms of antimicrobial class and potential vulnerabilities, and which can diversify our antimicrobial therapeutic portfolio [2].

Here, we further considered the properties of a historic antibiotic scaffold. Streptothricin was originally isolated by Waksman and Woodruff in 1942 from a soil *Actinomyces* [3]. It generated intense initial excitement as the first identified antibiotic natural product with potent activity against gram-negative organisms and additional activity against *Mycobacterium tuberculosis* [4]. It was specifically noted to completely cure *Brucella abortus* infection in guinea pigs [5] and otherwise lethal *Salmonella paratyphi* B infection in mice [6], infections of concern at the time. Based on these attributes, it went into fermentative production at Merck. However, in a limited human trial (details not published) [7], the streptothricin natural product induced reversible kidney toxicity, and, therefore, was not further pursued as a therapeutic.

**Fig 1:**
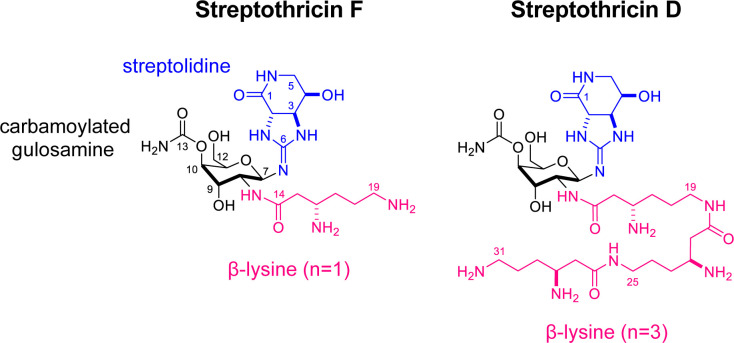
Structure of S-F and S-D. Streptothricins share streptolidine and carbamoylated gulosamine sugar moieties. They are distinguished by differing numbers of β-lysines attached end-to-end through amide bonds to the ε-amino groups. Nourseothricin is the natural product mixture of several streptothricins, predominantly S-F (1 β-lysine) and S-D (3 β-lysines). Acetylation of the β-amino group blocks activity and is the major known mechanism of antimicrobial resistance to streptothricins. S-D, streptothricin D; S-F, streptothricin-F.

Streptothricin is now known to be a natural product mixture, currently referred to as nourseothricin. For clarity, we will henceforth refer to the natural product mixture as nourseothricin and individual components by their specific streptothricin designation. Each streptothricin in nourseothricin consists of 3 linked components: (a) a streptolidine, a guanidine-lactam bicyclic 5–6 ring system; (b) a carbamoylated gulosamine; and (c) a homopolymer consisting of varying numbers of β-lysine residues, linked head to tail. The individual streptothricins are designated by letter suffixes (A-F and X), based on the number of lysine residues, with the most abundant streptothricins in the natural product, streptothricin F (S-F) and streptothricin D (S-D), having 1 and 3 lysine moieties, respectively (see Fig 1).

Initial studies on toxicity in animals from the 1940s used only semiquantitative activity measures and impure compound with large doses presumably exceeding 100 mg/kg [8] and are therefore problematic to interpret. More recent studies in mice with fractionated streptothricins showed that toxicity varies with the length of the poly-β-lysine chain: specifically, a murine LD_50_ of 300 mg/kg for S-F with 1 lysine compared to an LD_50_ of approximately 10 mg/kg for S-D and S-C with 3 and 4 β-lysines, respectively [8,9]. For comparison, the murine LD_50_ is 52 mg/kg IV for gentamicin [10]; 40 mg/kg intraperitoneally (IP) for colistin methanesulfonate [11]; and 260 mg/kg IP for tobramycin [12]. Streptothricin’s delayed toxicity towards renal proximal convoluted tubules, only demonstrated histologically in the literature for S-C in rats [13], has not been fully explained at a mechanistic level [8]. Of note, S-C at 40 mg/kg, well above the lethal dose, did not cause liver damage as assessed by serum aspartate aminotransferase (AST) and alanine aminotransferase (ALT) enzymatic assays [13], highlighting kidney damage as the primary limiting toxicity.

Studies 40 years ago using in vitro translation systems determined that S-F both inhibited prokaryotic translation and induced concentration-dependent ribosomal miscoding activity, similar to aminoglycosides such as gentamicin [14]. Notably, however, it did not inhibit translation in rat liver extracts, suggesting prokaryotic selectivity.

Based on potentially insufficiently explored therapeutic potential, we sought to further characterize the properties of S-F and S-D, the major constituents of nourseothricin, purified to homogeneity using modern techniques and authenticated by several complementary state-of-the-art methods. This analysis included in vitro and in vivo analysis and mechanism of action studies combining use of both mutational resistance and cryo-EM–based structural analysis. Taken together, our data establish S-F as a potently bactericidal natural product scaffold with a unique mechanism of action with what we believe are compelling properties for future medicinal chemistry exploration. To this end, we also separately established a diversity-enabling total synthesis for S-F, which is reported elsewhere [15].

## Results

### Activity spectrum studies

Our original studies with streptothricins were motivated by the observation that a nourseothricin resistance cassette served as a reliable genetic marker for experiments in otherwise highly drug-resistant gram-negative pathogens [16,17]. This led to the question of whether nourseothricin or its constituent streptothricin homologues had more general activity against multidrug-resistant gram-negative pathogens such as CRE and *A*. *baumannii*. We, therefore, adapted a previously reported method to purify S-F (1 β-lysine) and S-D (3 β-lysines) to homogeneity using Sephadex chromatography [9]. Confirmation of purity and precise molar content was determined by elemental analysis, NMR, and LC–MS as described in S1 Results; S1 Materials and Methods; S1–S3 Figs; and S1 and S2 Tables.

Using broth microdilution minimal inhibitory concentration (MIC) testing, we determined the MIC of S-F, S-D, and nourseothricin for a large group of multidrug-resistant CRE. These included strains expressing metallo- and/or serine carbapenemases and the pandrug-resistant, NDM-1–expressing *Klebsiella pneumoniae* Nevada strain, AR-0663 [18]. For these CRE (*n =* 39), MIC_50_ and MIC_90_ for S-F, S-D, and nourseothricin, respectively, were 2 and 4 (range 1 to 4) μM; 0.25 and 0.5 (range 0.25 to 2) μM; and 0.5 and 1 (range 0.25 to 2) μM. The average MIC was 5.6-fold greater for S-F than for S-D and 4.2-fold greater for S-F than for nourseothricin. We tested 4 *A*. *baumannii* strains in parallel with S-F, S-D, and nourseothricin (FDA-CDC #399–402) and found that the MIC for each was 4-fold greater for S-F than for S-D and nourseothricin. Data for individual isolates are listed in S3 Table. We also found that nourseothricin demonstrated broad activity against a diverse MDR/XDR *A*. *baumannii* strain set (*n =* 104), many isolates from wounded US soldiers [19]: MIC_50_ = 2 μM; MIC_90_ = 32 μM, MIC_99_ = 32 μM; range <0.125 to 64 μM.

An LC–MS analysis of commercially purchased nourseothricin showed a composition of 65.5% S-F, 29.6% S-D, and 4.9% S-E (2 β-lysines). Therefore, the somewhat enhanced activity of S-D (mean MIC 0.44 μM) compared with nourseothricin (mean MIC 0.58 μM) for CRE likely reflects a combinatorial effect of the more potent S-D and less potent S-F, as well as contributions from other minor streptothricin congeners in the nourseothricin natural product mixture.

### In vitro time-kill analysis

**Fig 2:**
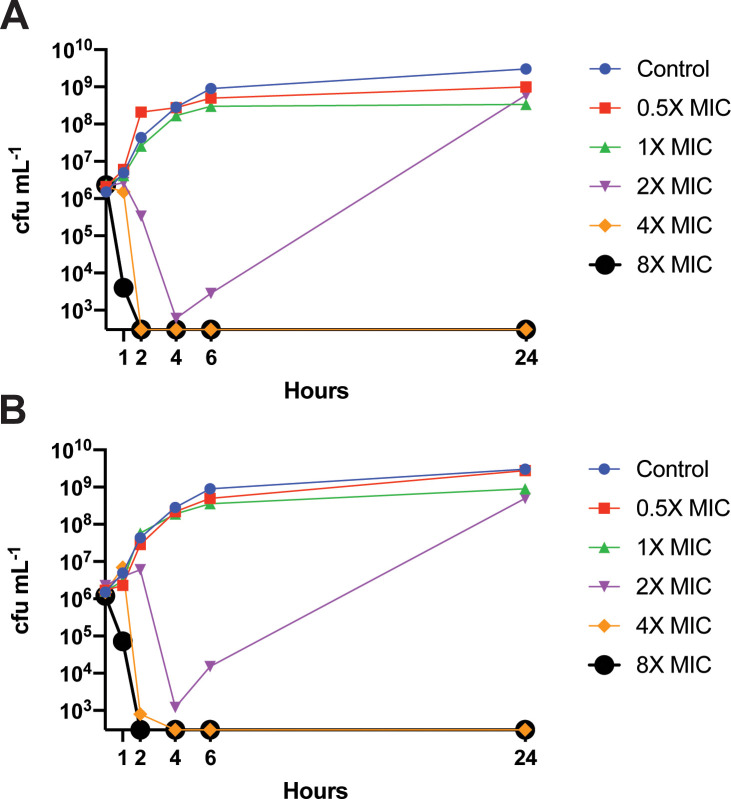
Rapid bactericidal activity against the *Klebsiella pneumoniae* Nevada strain. **(A)** Nourseothricin MIC 0.25 μM. **(B)** S-F MIC 1 μM. Data are available in S1 Data. MIC, minimal inhibitory concentration; S-F, streptothricin-F.

Bacterial killing by unfractionated, crude preparations of streptothricins was previously noted by Waksman and Woodruff for *Escherichia coli* and *Micrococcus lysodeikticus* after 17 and 65 hours of exposure without reference to an underlying MIC [3]. To characterize potential bactericidal activity, we therefore performed time-kill analysis for S-F and nourseothricin against the pandrug-resistant CRE *K*. *pneumoniae* Nevada isolate (AR-0636). Notably, we observed rapid bactericidal activity for both within 2 hours of exposure at 4X MIC without regrowth (Fig 2, S1 Data). Findings were similar for carbapenem-resistant *A*. *baumannii* isolate MSRN 1450 (S4 Fig, S2 Data). Therefore, bactericidal activity for highly resistant CRE and *A*. *baumannii* isolates were observed using current methods.

### In vitro translation assays

**Fig 3:**
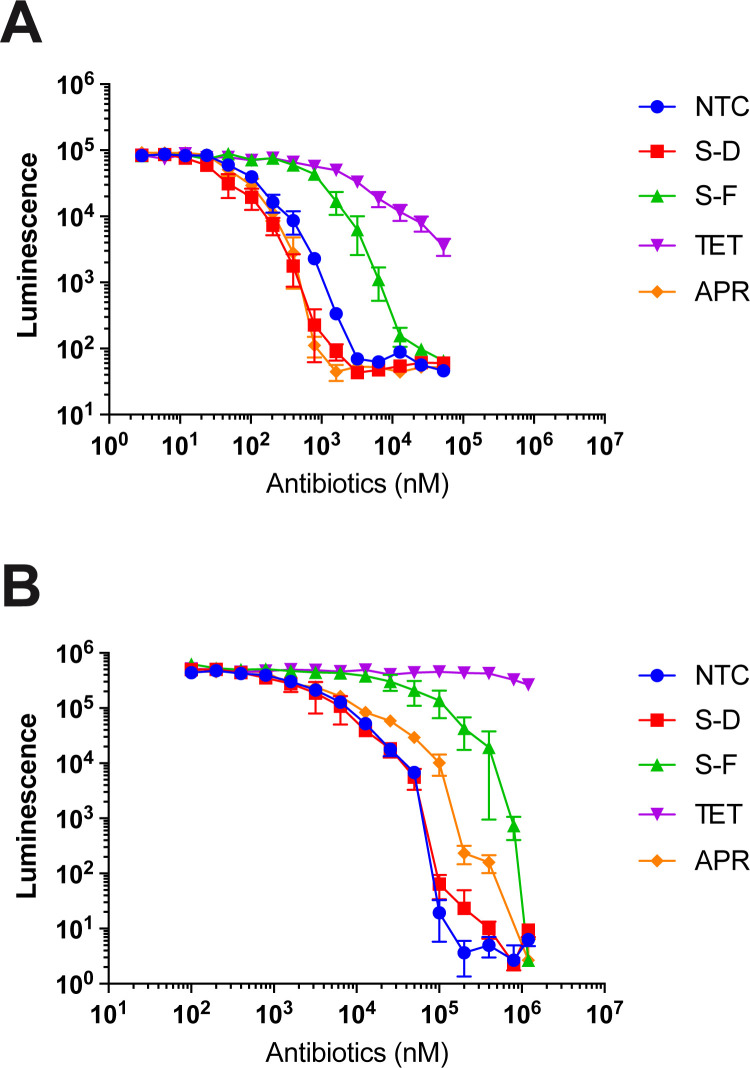
Inhibition of prokaryotic and eukaryotic translation. **(A)** Inhibition of prokaryotic in vitro translation using coupled in vitro transcription–translation extracts with readout from a nanoluciferase reporter. **(B)** Inhibition of eukaryotic in vitro translation using coupled in vitro transcription–translation extracts with readout from a nanoluciferase reporter. S-F, S-D, NTC, APR, and TET data represent mean and standard deviation from 3 independent experiments. Data are available in S3 Data. APR, apramycin; NTC, nourseothricin; S-D, streptothricin D; S-F, streptothricin-F; TET, tetracycline.

We measured in vitro action on ribosome translation (i.e., independent of bacterial permeability and efflux effects) using commercially (NEB) available prokaryotic (*E*. *coli*) and eukaryotic (rabbit reticulocyte) in vitro coupled transcription–translation systems with readout using nanoluciferase gene constructs (S5 Fig). In this analysis, we found that both S-F and S-D were approximately 40-fold more selective for prokaryotic than eukaryotic ribosomes based on IC_50_ values determined from Hill slopes of dose–response curves (Fig 3, S3 Data). Interestingly, S-D was approximately 10-fold more potent than S-F on a molar basis, helping to explain the lower MICs noted for the former. Inhibition by apramycin and tetracycline was as expected. As the difference in translational inhibition by S-F and S-D was greater than the difference in their mean MICs, we, therefore, considered whether S-F might have relatively enhanced bacterial penetrance. However, the relative activity of S-D and S-F was not changed in isogenic *E*. *coli lptD* and *tolC* mutants (S4 Table), suggesting that the outer membrane permeability and efflux pump activity, as assessed by these measures, did not contribute to this discrepancy in activity observed in extracts and whole bacteria.

### Cytotoxicity

**Fig 4:**
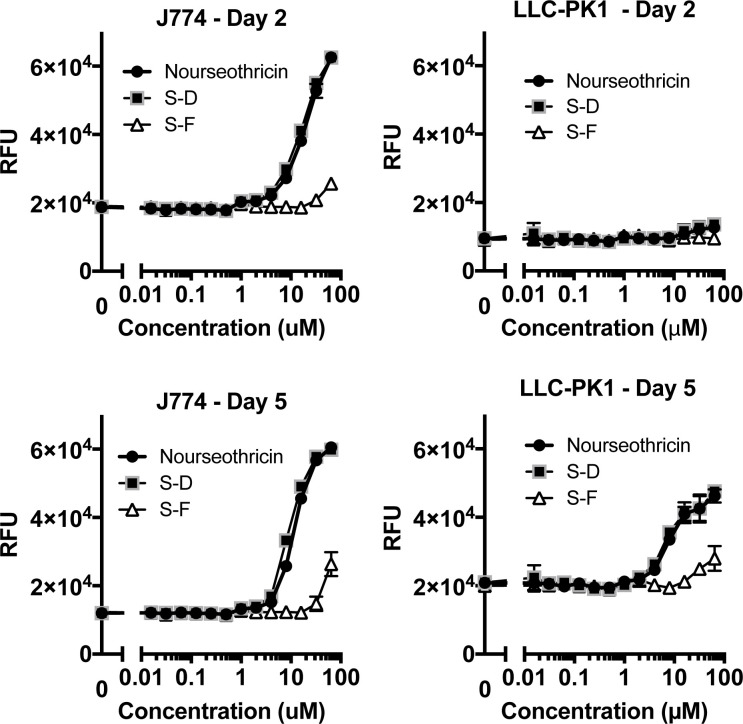
Cytotoxicity of streptothricins against mammalian cell lines. J774 macrophages and LLC-PK-1 renal epithelial cells were treated with 2-fold doubling dilutions of nourseothricin, S-D, and S-F for up to 5 days in the presence of SYTOX-Green. SYTOX-Green is a cell membrane-impermeant nucleic acid binding dye that fluoresces on binding to nuclear DNA. It therefore provides a real-time readout of eukaryotic cell membrane permeabilization associated with cell death that can be continuously monitored through fluorescence measurements. Cytotoxicity was minimal to absent after a single day incubation but increased on subsequent days. Nourseothricin and S-D effects were essentially indistinguishable. S-F toxicity was only observed at molar concentration at least 10-fold greater than S-D beginning at 32 μM, significantly above MIC ranges observed in activity spectrum analysis. Each data point represents mean and standard deviation for assays performed in quadruplicate. Data are available in S4 Data. MIC, minimal inhibitory concentration; S-D, streptothricin D; S-F, streptothricin-F.

Eukaryotic cell cytotoxicity was determined against J774A.1 macrophage and LLC-PK1 proximal tubule kidney cells line during a 5-day incubation. Cytotoxicity was measured using a previously established SYTOX Green exclusion, real-time fluorescence assay [20,21]. Notably CC_50_ for these cell lines was >10× higher for S-F than S-D (Fig 4, S4 Data). Cytotoxicity was also relatively delayed for S-F compared with S-D, first apparent on day 2 of incubation, with more prominent cytotoxicity for both S-F and S-D noted after 5 days of incubation. The delay in observed cytotoxicity was more pronounced for LLC-PK-1 in which cytotoxicity for S-D and S-F were not appreciable until day 3, and of borderline magnitude for S-F at day 5 (Fig 4). Notably, the lowest observable adverse effect level for S-F during prolonged 5-day incubation was 32 μM, substantially above the MIC_99_ of 4 μM for CRE.

### Maximum tolerated dose

**Fig 5:**
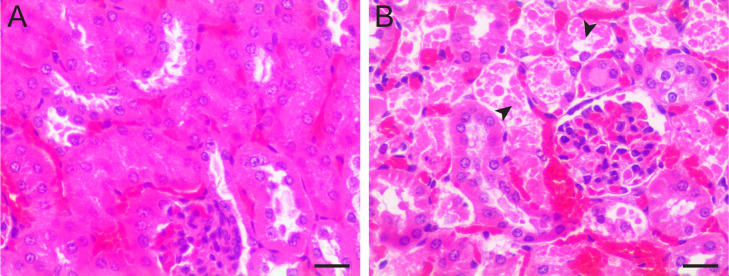
Delayed nephrotoxicity occurs at >10-fold higher doses of S-F than nourseothricin. **(A)** S-F dosing at 100 mg/kg without obvious histological abnormality in kidney. **(B)** Nourseothricin dosing at 10 mg/kg showing cellular necrosis and nuclear degeneration of proximal convoluted tubule epithelial cells (arrowheads). Glomeruli and distal tubules were spared. Tissue was harvested 3 days after dosing. Size bar = 20 μM.

To identify the single-dose maximum tolerated dose (MTD) of S-F and nourseothricin, CD-1 mice were injected IP with ascending doses of 0, 50, 100, 200, and 400 mg/kg S-F and 5, 10, 20, and 50 mg/kg nourseothricin, respectively (*n =* 4 per dose). Nourseothricin was used as a surrogate for S-D, based on the difficulty of isolation of the latter in quantities needed for the experiments. Over the next 72 hours, no signs of distress were observed with dosing up to 200 mg/kg of S-F and 20 mg/kg of nourseothricin, respectively. Two mice at 400 mg/kg of S-F and 1 mouse at 50 mg/kg nourseothricin died, or became moribund and were euthanized. Histology of kidneys was specifically examined for all mice based on described delayed renal toxicity in rats for the streptothricin natural product [13]. For S-F, proximal convoluted tubular damage was noted sporadically at 100 mg/kg and more diffusely at ≥200 mg/kg. For nourseothricin, proximal tubular damage was pronounced at ≥10 mg/kg. Glomeruli, distal tubules, and vasculature were spared (Fig 5). Therefore, the maximum dose without observed pathological effect was 50 mg/kg for S-F and 5 mg/kg for nourseothricin, respectively.

### Murine thigh infection model

**Fig 6:**
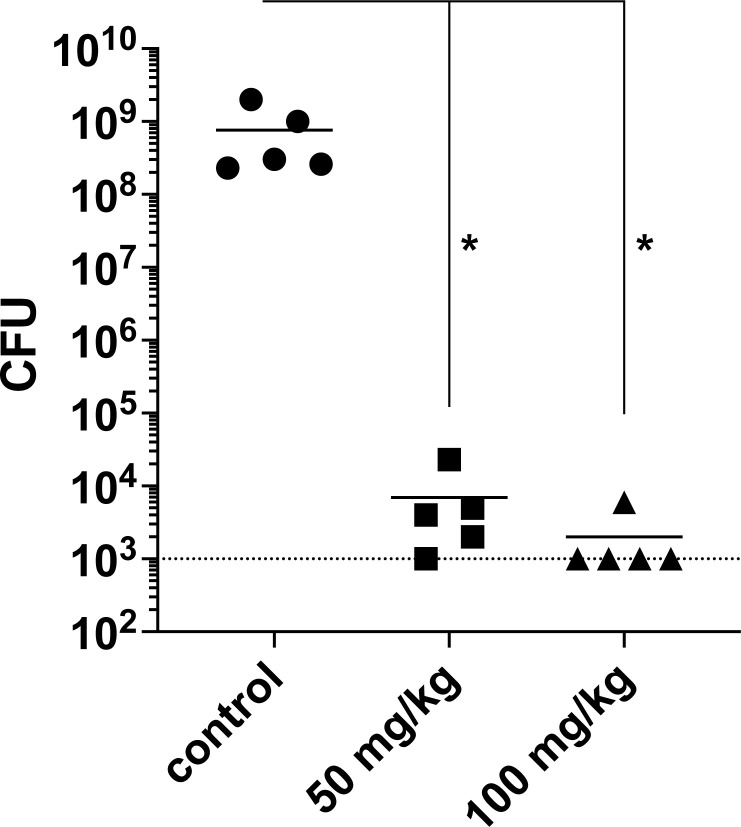
Murine thigh infection model. S-F demonstrated substantial therapeutic effect against pandrug-resistant *Klebsiella pneumoniae* Nevada AR-0636 at doses without observable or minimal toxicity. Dotted line is assay limit of detection. Data points for 5 mice per condition are shown. * designates significant difference from untreated controls using Kruskall–Wallis nonparametric test. Data are available in S5 Data.

Therapeutic effect after a single dose of S-F was tested in the murine thigh model after infection with the pandrug-resistant Nevada CRE strain (AR-0636). For these studies, neutropenic mice were rendered mildly renal deficient with uranyl nitrate to more closely simulate human excretion kinetics [22]. Tissue was harvested 24 hours postinfection. In the absence of treatment, CFU increased >2log_10_. In contrast, S-F at 50 mg/kg or 100 mg/kg led to a greater than 5log_10_ reduction in CFU with absence of detectable CFU in 3 of 5 mice treated with the higher dose (Fig 6, S5 Data). Thus, substantial in vivo bactericidal treatment effect was observed with a single dose of S-F at levels with minimal or no observable toxicity.

### Resistance studies suggest novel target

To identify potentially unique interactions with the ribosome, we sought to identify high-level nourseothricin resistance mutants by selecting for growth on media containing nourseothricin at 32 to 64 times the MIC. Two classes of high-level nourseothricin resistance mutants were obtained only in the single *rrn* operon, SQ110, *E*. *coli* mutant strain at a frequency of approximately 6.2 × 10^−9^ but not in wild-type (wt) *E*. *coli*, which has 7 *rrn* operons, implying mutations were recessive. In total, 54, 2, and 5 independently isolated strains containing 16S rRNA C1054A, A1196C, and A1196G mutations, respectively, were identified. All grew noticeably slower than the SQ110 parent. In addition, 16S rRNA C1054T was identified in 16 strains that proved nonviable on passaging from frozen stocks and were therefore not further analyzed. In Table 1, we show modal MIC values from 3 biological replicates for 2 randomly selected mutants of each type to rule out potential contributions of secondary mutations. C1054 and A1196 mutants conferred >256-fold and 16- to 32-fold increases in MIC for nourseothricin, respectively. Linkage of these mutations to the resistance phenotype and confirmation that these mutations are recessive were further supported by full restoration of susceptibility after introduction of a wt *rrnB* rRNA operon into the mutant strains, in contrast to absence of effect with the empty vector control (S5 Table). Furthermore, in vitro translation extracts prepared from the SQ110 C1054A mutant strain N1 were resistant to nourseothricin at the highest concentrations tested (S6 Fig and S6 Data). In contrast, extracts from parent SQ110 were susceptible with inhibition similar to observations with commercial *E*. *coli* K-12 extracts (Figs 3 and S6). Confirming that the resistance observed with the C1054A extract was nourseothricin specific, in vitro translation extracts from both parent SQ110 and strain N1 C1054A were similarly inhibited by the positive control, apramycin.

**Table 1:** Modal MIC values for resistance mutants identified in single rrn operon *E*. *coli* strain identifies unique target in helix 34 of ribosomal 16S rRNA.

strain	SQ110	N1	N8	N9	N11	N12	N13
genotype	wt	C1054A	C1054A	A1196G	A1196G	A1196C	A1196C
**nourseothricin**	1	>256	>256	32**	16*	64	64
**tetracycline**	2	4	8	4	2	4*	4*
**doxycycline**	2	8	8	4	4	2*	2
**minocycline**	2	16	8	4	4	4*	4
**tigecycline**	0.5	0.5	0.5	0.25	0.25	0.25	0.25
**eravaycline**	0.125	0.125	0.125	0.125	0.125	0.125	0.125

SQ110 contains only a single *rrn* operon. Spontaneous nourseothricin SQ110 resistance mutants were selected for by plating on high concentrations of nourseothricin. Mutations in 16S rRNA identified in these resistant strains are listed under genotype. MIC values are in μg/mL determined after 40 hours of incubation, as single ribosomal RNA strains, especially mutants, grew slowly, and differences between growth and inhibition could not be reliably distinguished at earlier time points. Modal MIC values from at least 3 biological replicates are shown. Unless otherwise indicated, MIC range was ≤2 doubling dilutions.

*The range of MICs obtained varied over 3 doubling dilutions.

**MIC range was 32–256 μg/mL in 4 biological replicates.

Despite overlap of identified nourseothricin resistance mutations with the reported binding pocket of tetracyclines in 16S rRNA helix 34 [23], biological data suggested that the mechanism of action of the bactericidal, miscoding-inducing streptothricin and bacteriostatic tetracyclines should be distinct. Supporting this notion, C1054A only conferred modest cross-resistance, and A1196C/G conferred negligible if any cross-resistance to tetracycline, doxycycline, minocycline, tigecycline, or eravacycline (Table 1), respectively. In addition, expression of the ribosomal protection protein, TetM, had no effect on activity of either nourseothricin or the apramycin control (known to bind helix 44), while conferring expected high-level resistance to tetracycline and minocycline (Table 2). Furthermore, a limited sampling of *Staphylococcus aureus* clinical strains expressing *tetM* gene, showed low nourseothricin MICs (0.5 μg/mL), while demonstrated the expected high MICs for tetracycline (S6 Table).

**Table 2:** TetM ribosomal protection protein does not interfere with nourseothricin activity.

	Apramycin	Tetracycline	Minocycline	Nourseothricin
pBad*LSSOrange*	1	0.5	0.25	0.25
pBad*TetM*^a^	1	64	64	0.5

^**a**^*tetM* from *Staphylococcus aureus* was cloned under an arabinose inducible promoter. As a control, an unrelated LSSOrange fluorescent protein was expressed from the same construct. Expression was induced by 66 mM arabinose. Data are the modal values for triplicate MIC measurements in μg/mL and are representative of data from multiple independent clones of tetM and different levels of arabinose induction.

### Cryo-EM characterization of streptothricins binding to the *A*. *baumannii* 70S ribosome

**Fig 7:**
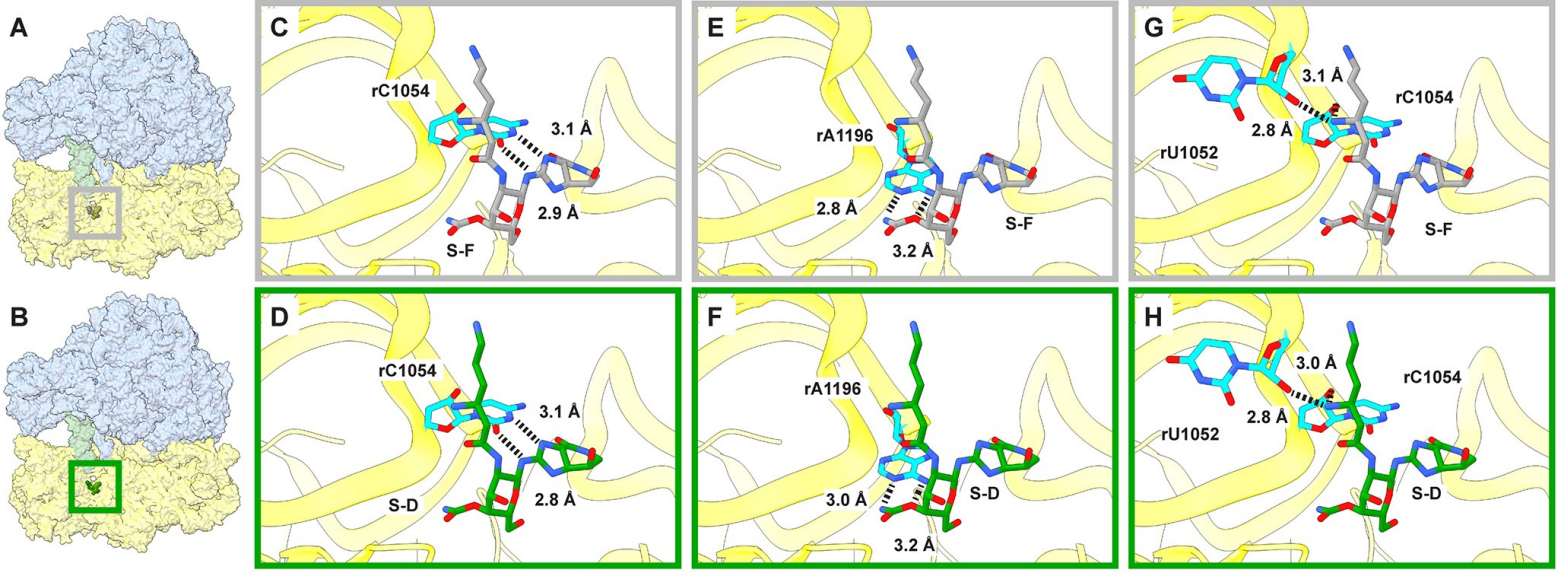
S-F and S-D binding sites in the ribosome. **(A, B)** Location of the S-F and S-D binding sites in the *A*. *baumannii* 70S ribosome with P-site tRNA, respectively. **(C, D)** Hydrogen-bonding interactions between the streptolidine moieties of S-F and S-D and C1054 of the 16S rRNA, respectively **(E, F)** Electrostatic interactions of the S-F and S-D 12-carbamoylated gulosamine moiety with A1196, respectively. **(G, H)** Electrostatic interactions between β-amino of the β-lysine and the O2’ atoms of C1054 and U1052. Throughout the figure, the 50S is blue, 30S is yellow, S-F is gray sticks, S-D is green sticks, and highlighted residues are cyan. The cryo-EM densities corresponding to the 2 terminal β-lysine moieties of S-D were very weak, indicating that these 2 lysine moieties are flexible. We therefore did not include these 2 terminal β-lysine moieties in panels D, F, and H. cryo-EM, cryo-electron microscopy; S-D, streptothricin D; S-F, streptothricin-F.

The structures of S-F and S-D bound to the 70S ribosome of *A*. *baumannii* were solved using single-particle cryo-electron microscopy (cryo-EM) (S7–S10 Figs, S7 and S8 Tables, S7 and S8 Data). Binding of S-F and S-D were only observed in cryo-EM structures following incubation of ribosomes with 100 μM of each antibiotic, but not when ribosomes were soaked with a physiologically more relevant 10 μM concentration. Throughout the 70S, 2 distinct S-F-binding sites were identified for 70S–S-F, while a total of 12 S-D-binding sites were observed for the 70S–S-D complex (S11 and S12 Figs). Notably, through comparison of both cryo-EM structures, only 1 S-F-binding site (site 1 of S11 Fig) was found to completely overlap with one of the S-D-binding sites (site 12 of S12 Fig). This site is located at the 30S head, specifically helix 34 of the 16S rRNA (Fig 7A and 7B). It is characterized by multiple hydrogen bonds between the streptolidine moiety of the streptothricin backbone and the 16S rRNA C-1054 nucleobase (*E*. *coli* rather than *A*. *baumannii* 16S rRNA base numbering is used; Fig 7C and 7D) and by the gulosamine sugar and in particular the C-10-carbamoyl moiety with the A1196 base (Fig 7E and 7F). The β-amino group of the β-lysine also interacts electrostatically with O2’ of the riboses in U1052 and C1054 (Fig 7G and 7H). A further polar contact was made between the C-10-carbamoyl moiety and the carbonyl group of the rProtein s3 E161 amide bond. Surprisingly, the cryo-EM densities corresponding to the 2 terminal β-lysine moieties of S-D were very weak, indicating that these 2 lysine moieties are flexible. We therefore did not include these 2 terminal β-lysine moieties in the 70S–S-D cryo-EM structure. It was observed that both S-F and S-D utilize a similar mode of binding to interact with the 70S ribosome, but this binding mode is quite distinct from other antibiotics, such as eravacycline and negamycin, which are known to bind to this region (S13 Fig).

The second S-F-binding site is found to partially overlap with site 2 of S-D (S11 and S12 Figs). This second S-F site is located at the proximity of the E-site in the 50S ribosomal subunit (S11A, S11C and S12B2 Figs). The streptolidine and gulosamine moieties of S-F and S-D overlap at this site, interacting with H20 of rProtein L28, while the interactions of the β-lysine tails differ. Binding by these streptothricins is occluded by binding of tRNA at the 50S E-site (S11B Fig).

While not seen in S-F, site 4 of S-D is located in the peptide exit tunnel (S12B Fig). The S-D molecule is stabilized via electrostatic interactions with A1612 of the 23S rRNA and residues K90, R92, A93, and R95 of rProtein L22. This site is in proximity of the binding site for macrolide antibiotics, which also occupy the peptide exit tunnel to interfere with peptide elongation. While these sites are not seen in the mutational experiments, sites 2 and 4 are located in vital locations of the ribosome, where the first is located in a position that may interfere with the transfer of deacylated tRNA to the E-site of the 50S and the second with the elongating peptide chain. These sites invite further experimentation. The remaining of the 9 other S-D binding sites (sites 1, 3, 5, 6, 7, 8, 9, 10, and 11) are shown in S12 Fig. As the 70S ribosome is a large complex, it is presumed to offer many binding sites that are potentially druggable. Our 70S–S-D structure indeed underscores the possibility that we can identify potential therapeutic strategies via this structural information. Of note, the orientation of A1492 and A1493, residues fixed in a flipped-out position during binding of 2-deoxystreptamine aminoglycosides and thought to stabilize binding of noncognate tRNAs [24], was not affected.

## Discussion

We demonstrate that streptothricins, in particular S-F, S-D, and the natural product mixture, nourseothricin, are highly active against contemporary, carbapenem-resistant *E*. *coli*, *Klebsiella*, *Enterobacter*, and *A*. *baumannii*. Their activity spectra are therefore highly relevant to the emerging gap in gram-negative antimicrobial coverage, even more so since they appear to act in a unique manor to kill bacterial cells.

Most of the existing description of streptothricin class antibiotic activity was from several decades ago and made use of unstandardized microbiological and physicochemical methods. In general, the natural product mixture was characterized as a whole without quantitative delineation of contributions from constituent streptothricins. Instead, here, we made use of modern preparative techniques and methods to characterize the activity of S-F and S-D in comparison with the natural product mixture currently available, gaining valuable insights into their respective properties.

We found that S-F maintained potent antimicrobial activity, although it was about 6-fold less potent than S-D. Importantly, S-F showed at least 10-fold lower toxicity than S-D and the natural product mixture, nourseothricin, in vitro and in vivo. Notably, in single, maximal tolerated dose experiments, treatment with the nourseothricin natural product mixture was associated with proximal tubule kidney damage at relatively low doses (10 mg/kg). In contrast, S-F showed a favorable therapeutic ratio with substantial therapeutic effect at 50 to 100 mg/kg to the point of single-dose sterilization of CRE infection with minimal or no renal histopathological toxicity.

Biologically, streptothricins demonstrate activity similar to 2-deoxystreptamine (2-DOS) containing aminoglycosides such as gentamicin, amikacin, tobramycin, and apramycin, both inhibiting protein synthesis with rapid, potent bactericidal activity [25], as observed here for CRE and *A*. *baumannii*, and inducing translational miscoding [14,26]. It was therefore an original expectation that streptothricins would interact with the 30S ribosomal subunit target of aminoglycosides in a similar manner. However, the combination of structural and resistance studies supported an alternative and unique mechanism of action.

The 2-DOS aminoglycosides are known to bind helix 44 in 16S rRNA (residues 1400 to 1410 and 1490 to 1500), forming a binding pocket near the ribosome decoding center [27–31] (S14C Fig). In doing so, they induce a flipping out of 16S rRNA bases, A1492 and A1493. This molecular rearrangement is either associated with or stabilizes binding of noncognate tRNA to the A-site leading to miscoding [32]. Consistent with their binding profile, the activity of 2-DOS aminoglycosides is blocked by methylation of residues G1405 and A1408, as well as by recessive, resistance-conferring point mutations at positions 1406, 1408, 1409, 1491, and 1495 [33–39]. However, despite demonstrating miscoding and rapid bactericidal activity, characteristics of aminoglycosides, neither methylation of G1405 and A1408 by ArmA and NpmA resistance enzymes (S9 Table), nor the A1408G and G1491A mutants (S10 Table) tested, affected nourseothricin activity (S1 Results).

Instead, our structural and resistance studies identified an interaction with helix 34 distinct from other known translation inhibitors. Specifically, S-F makes direct polar contacts with nucleobases, C-1054 and A-1196. This contrasts with the stacking interactions of tetracyclines [40] with C-1054 (S13 Fig) and interaction of the pseudodeptide antibiotic, negamycin, solely with phosphoribose backbone of helix 34 (S13 Fig) [41,42]. Of interest, bonding of streptolidine with C1054 (S14A Fig) resembles G:C Watson–Crick base pairing (S14B Fig). This observation likely accounts for high-level resistance conferred by nucleobase changes, which would interrupt this strong bonding interaction, and therefore cannot be tolerated. Similarly, 2 hydrogen bonds between the carbamylated gulosamine and the A1196 nucleobase likely account for the midrange resistance levels associated with mutation at this position. In contrast to the strong effects on streptothricin activity, C1054A and A1196C/G mutations had negligible effect on tetracycline, doxycycline, minocycline, tigecycline, and eravacycline MIC values. In addition, the streptothricin resistance mutations were distinct from previously isolated mutations conferring strong resistance to tetracyclines, at 16S rRNA U1060A and G1058C [43–45]; spectinomycin at A1191 and C1192 [31,33,39,46,47]; and negamycin, at U1060A, U1052G, A1197U [41,42], consistent with known contacts in structural studies, and further supporting a distinct biological mechanism of action. Lastly, the ribosomal protection protein, TetM, which blocks access to the tetracycline binding site [48], also had no effect (Table 2). Structural studies indicated that P591 in loop 3 of domain IV of TetM makes stacking interactions with C1054, similar to tetracyclines. However, superimposition of the S-F and Tet-M ribosome structures suggest lack of steric clash of TetM with the S-F/S-D binding site, providing a structural rationale for TetM’s lack of effect on streptothricin activity (see S15 Fig).

Our structural data further explain streptothricin acetyl transferase-based resistance to S-F and S-D associated with acetylation of the β-amino group of the first β-lysine attached to the gulosamine sugar. Specifically, our modeling indicates that acetylation cannot be accommodated in the S-F binding pocket without steric clash with the phosphoribose backbone of U1052 and C1054 (Fig 7F). Finally, our data help explain several observations related to activity of naturally occurring S-F analogs. In S-F, the hydrogens on C-2 and C-3 of the streptolidine β-lactam ring are located in *trans* (2S, 3S configuration). Although comparative analysis between naturally occurring S-F analogs has not been systematically performed, an S-D analog with a *cis*-fused streptolidine lactam moiety (2R, 3S) showed a 32-fold increase in MIC for *B*. *subtilis* [49] compared to a previous report for S-F by the same group [50]. The disruption of the stereochemistry at the interface of the critical binding interactions of the streptolidine and C-1054 presumably accounts for this loss of activity, analogous to the disruption of this interaction in the C1054A mutant. In contrast, methylation of the nitrogen in the streptolidine lactam ring, with, in the analog, albothricin [51], or without, in the analogs, A37812 [52] and N-methyl S-D [53], removal of the C-4 hydroxyl group from the streptolidine ring, both positions outside of the interface with C1054, did not appear to undermine activity. Furthermore, carbamoylation at the C-12 (see Fig 1) rather than C-10 position of the gulosamine ring (analog A-269A’) was associated with an approximately 16× increase in MIC [50], presumably because of disruption of contacts with A1196, analogous to the disruption of the same interactions in the A1196C/G mutants (Table 1). The amino terminus (ε-amino group) of the β-lysine appears unbonded and surface exposed, allowing for the presumably relatively unrestricted addition of additional β-lysines, e.g., in S-D. Interestingly, substitution of the β-lysine with the α-alpha amino acids, methyl-glycine (named alternatively A-269A [54], SF-701 [55], or LLBL-136 [56]), or methyl-imino glycine (named BD-12 [57] or LL-AB664 [58]), were tolerated and may even have superior activity. Modeling suggests new contact of the α-amino group of these amino acids with the C1054 ribose, replacing the contacts of the β-lysine β-amino group with the C1054 phosphate and U1052 ribose. Intriguingly, it is possible that existing streptothricin acetyltransferases may be inactive against these α-amino acids substitutions, an area of investigation not yet reported in the literature, and, therefore, these naturally occurring modifications might extend activity spectrum to otherwise resistant organisms.

Regarding potential mechanisms of action, previous structural studies have described stacking interactions between C1054 and the wobble nucleotide of the A-site tRNA, anti-codon stem loop [59]. Notably, superposition of the S-F structure onto C1054 in the PDB structures of *E*. *coli* (PDB 7K00 [60]) and *T*. *thermophilus* (PDB 4V8B [61], 3T1H [62], 1IBM [24], 4WQ1 [63], 6XHV [61], and 4V8B [64]) ribosome structures including various A-site tRNA and mRNA codons suggests steric interaction with the wobble nucleotide of the anti-stem loop structure (S14C Fig) and potential probing of the minor groove between the codon and anticodon. Intriguingly, too, C1054A was the first known ribosomal suppressor mutation of the UGA stop codon [65–68] and is associated with readthrough of all 3 stop codons [66], supporting a critical role of this nucleobase in coding fidelity. These observations lead to a hypothetical mechanism explaining both observed miscoding and translation inhibition by S-F through effects on tRNA binding to the A-decoding site. Specifically, S-F may directly interact with the third, wobble position, and stabilize noncognate A-site tRNA interactions, a mechanism of action previously proposed for negamycin [69]. It should be noted that we have thus far not solved structures with streptothricins bound to translationally active ribosome complexes including both A-site and P-site tRNAs and mRNA. These complexes would provide further insights into mechanism of action.

It was theorized previously that the observed concentration dependence of S-F induced miscoding might be a consequence of multiple binding sites with presumably synergistic or additive mechanisms of action [14]. However, the other binding sites of S-F and S-D identified in cryo-EM structures in the 70S ribosome appear not to confer significant antimicrobial activity on its own, based on minimal, if any, retained activity when the 30S binding site is disrupted in the C1054A mutant. Nevertheless, these results do not rule out contribution to antibacterial activity once the primary 30S binding site is engaged. Notably, the supraphysiological 100 μM concentration of streptothricin used for cryo-EM studies (streptothricins were not resolved when soaking at 10 μM concentrations) likely contributed to the numerous additional and presumably nonspecific and physiologically irrelevant secondary binding sites observed.

Of interest, the nucleotides corresponding to 16S rRNA C1054 and A1196 in human 18S rRNA (PDB 7TQL; [70]) are conserved and occupy similar flipped out, stacked positions in helix 34 (S16 Fig). Likewise, the bacterial G530 from helix 18, which is also predicted to make contact with the streptolidine ring on the side opposite of its interaction with helix 34, and its human counterpart occupy nearly identical positions. Although bacterial s3.E161 is replaced by s3.Q145 in the human s3 rProtein, the position of their respective amide bond C-terminal carbonyl groups, which is predicted to make polar contact with the streptothricin C-10-carbamoyl moiety, is conserved. Therefore, it is not clear what accounts for the relative selective inhibition of prokaryotic translation. It is possible that the sum of small deviations in these respective structures and others not appreciated contribute to this selectivity. Solving structures of streptothricins bound to the human ribosome may help address some of these possibilities.

In summary, we present data for compelling bactericidal activity of S-F against contemporary multidrug-resistant CRE and *A*. *baumannii* pathogens with in vivo confirmation of efficacy against an emblem of gram-negative antibiotic resistance. We also find evidence for unique mechanism of action targeting 16S rRNA helix-34 distinct from other known translation inhibitors. We therefore believe that further early-stage exploration of the historic scaffold is warranted with the ultimate goal of identification of analogs with potential for therapeutic development.

## Materials and methods

### Microbial strains

CRE isolates were from the FDA-CDC Antimicrobial Resistance Isolate Bank (AR-Bank) including the Nevada Strain (AR-0636) and the BIDMC clinical collection sequenced as part of the CRE genomics project [71]. *A*. *baumannii* isolates were from the AR-Bank and the Multidrug Resistant Organism Repository and Surveillance Network (MRSN) at the Walter Reed Army Institute of Research (WRAIR). Strains are listed in S3 Table. The single ribosomal operon strain, *E*. *coli* SQ110, was from the Coli Genetics Stock Center (Yale University, New Haven, CT) and was originally described by Squire and colleagues [72].

### Streptothricin purification

Purification of separate streptothricin compounds from nourseothricin sulfate (Gold Biotechnology, St. Louis, MO) was performed through modification of a previously reported method [9]. A glass column (150 cm × 2.4 cm) was packed with Sephadex LH-20 size exclusion gel (GE Healthcare, Chicago, IL) using a mobile phase of 10% methanol/H_2_O. The flow rate was adjusted using compressed air to 0.6 mL/min. Purifications were run in batches of approximately 300 mg of nourseothricin sulfate, which was diluted in 0.6 mL of H_2_O and loaded dropwise directly onto the top of the column. A mobile phase of 10% methanol/H_2_O was used for elution and fraction sizes of 3 mL were collected. Fractions testing positive for the ninhydrin stain were analyzed for purity by LC–MS. Pure S-D began eluting after approximately 120 mL of mobile phase, followed by mixed fractions of streptothricin D/E/F, and, finally, pure streptothricin F. Pure fractions for S-D were combined, frozen, and lyophilized to give a powdery, off-white solid. Pure fractions for S-F were combined, frozen, and lyophilized to give a powdery, off-white solid. Additional experimental details including ^1^H- and ^13^C-NMR spectra, mass spectrometry, as well as elemental analysis data of S-F and S-D can be found in the supporting information.

### MIC testing

The Clinical Laboratory and Standards Institute (CLSI) broth microdilution reference method was used for MIC testing [73]. Bacterial inocula were prepared by passaging previously frozen bacterial strains on sheep blood agar plate, culturing for 18 to 24 hours, except where noted, and suspending isolated colonies to 0.5 McFarland (approximately 1 × 10^8^ CFU/mL) in 0.9% NaCl solution. This suspension was diluted 1:300 into cation-adjusted Mueller–Hinton broth (BD Diagnostics, Franklin Lakes, NJ) to achieve a final inoculum concentration of approximately 5 × 10^5^ CFU/mL in 100 μL well volumes in round-bottom, 96-well plates (Evergreen Scientific, Los Angeles, CA). Doubling dilutions or antimicrobials were dispensed using the D300 digital dispensing method for final doubling dilution concentrations ranging from 0.125 to 256 μg/mL [74–76]. The inkjet printing methodology was previously extensively validated and found to be as accurate and more precise than manual dilution techniques described in CLSI methodology [76]. MIC values were determined after incubation for 16 to 20 hours.

### Time-kill studies

Time-kill studies were performed as previously described [77]. At time 0, antibiotics and inoculum were combined in cation-adjusted Mueller–Hinton broth (BD Diagnostics, Franklin Lakes, NJ) at various antibiotic concentrations based on multiples of each isolate’s MIC as determined through broth microdilution assays. Each tube was incubated at 35°C in ambient air on a platform shaker. Sterility, treatment, and no antibiotic growth controls were plated at 0-, 1-, 2-, 4-, 6-, and 24-hour time points, respectively. At each time point, the number of viable bacteria were quantified using the drop plate method [78] as a substitution for the CLSI spread plate method. Specifically, 10 μL drops of serial 10-fold serial dilutions of bacterial suspension were plated. As previously described [78], only drops with 3 to 30 colonies were counted. The limit of detection for this assay was conservatively set at 300 CFU mL^−1^ taking into account Poisson distribution considerations.

### In vitro translation assays

Prokaryotic and eukaryotic nanoluciferase reporter constructs were constructed as follows: For the bacterial nanoluciferase (Nluc) reporter construct, a DNA fragment containing a 516-bp fragment from pNL1.1 Promega, (Madison, WI), including the NLuc open reading frame and Shine-Delgarno sequence and an upstream T7 promoter sequence (TAATACGACTCACTATAGG) was synthesized as a gBlock by IDT (Integrated DNA Technologies, Coralville, IW). The T7_Nluc fragment was then amplified using primer pairs, T7Nluc-F and T7NLuc-R (S11 Table), and ligated into the pCR XL TOPO vector (Invitrogen, Carlsbad, CA) to create pCR XL TOPO-T7NLuc (S5A Fig).

For the eukaryotic Nluc reporter construct, the Nluc open reading frame without the methionine initiation codon was amplified with primers, Nluc_BamHI-F and Nluc_PstI-R (S11 Table). The amplicon was digested with BamHI and PstI, cloned into similarly digested pT7CFE1-NFTag (#88865, Thermo Fisher, Waltham, MA) in frame with leading sequence and downstream of the vector IRES and T7 promoter sequences to create pT7CFE1-NLuc (S5B Fig).

Nourseothricin, S-D, and S-F were distributed to a 384-well black microplates (PHENIX Research, Swedesboro, NJ). For bacterial in vitro translation assays, *E*. *coli* S30 Extract System for Circular DNA (Promega, #L1020) and 100 ng of pCR XL TOPO-T7NLuc were then added to each reaction well, making use of the upstream vector, pLac promoter rather than the cloned T7 promoter for expression in the current studies. After incubation at 37°C for 60 minutes, an equal volume of PBS buffer with 1 μg/mL Nluc-specific furimazine substrate (AOBIOUS, Gloucester, MA) was added to each well, and luminescent signal was detected with an Infinite M1000 Pro (TECAN, Morrisville, NC) plate reader. Eukaryotic in vitro translation was performed with the TnT-T7 Quick Coupled Transcription/Translation System (# L1170, Promega). TnT master mix, 100 ng of pT7CFE1-NLuc plasmid DNA, and methionine were added to test wells per manufacturer’s instructions. After incubation at 30°C for 80 minutes, Nluc signal was detected as described above.

### Eukaryotic cell cytotoxicity

J774A.1 mouse macrophage and LLC-PK1 porcine renal tubule epithelial cell lines were plated in 384-well tissue culture dishes with white walls and clear bottoms (Greiner, Monroe, NC, catalog #781098) at approximately 7.0 × 10^5^ cells/cm^2^ and approximately 7.0 × 10^4^ cells/cm^2^, respectively, in M199 medium lacking phenol red supplemented with 3% porcine serum and a final concentration of 125 nM SYTOX Green. Approximately 1 day after plating (at 50% to 75% confluence), the cell lines were treated with 2-fold doubling dilutions of nourseothricin (Gold Biotechnology), S-D, and S-F, dispensed with the HP D300 digital dispensing system (HP, Palo Alto, CA) using Tween-20 as the surfactant. Microplates were vortexed for 30 seconds to ensure thorough mixing within the wells prior to incubation. SYTOX Green fluorescence was read with a TECAN M1000 multi-mode reader using excitation 485 ± 7 nm and emission 535 ± 10 nm settings at indicated time points with otherwise continuous incubation at 37°C with 5% CO_2_ for 5 days.

### Maximum tolerated dose (MTD) determination

CD-1 (ICR) female mice weighing 25 to 30 g each were purchased from Charles River Laboratories (Kingston, NY). Mice were injected IP with 0, 10, 20, 50, 100, 200, and 400 mg kg^−1^ of purified streptothricin-F or nourseothricin. On day 3 post dosing, mice were euthanized and kidneys fixed in 10% neutral phosphate-buffered formalin overnight, embedded in paraffin, and 10 μm tissue sections stained with hematoxylin and eosin at an institutional core facility using standard procedures as described previously [79].

### Neutropenic mouse thigh model

Mice were pretreated with cyclophosphamide and uranyl acetate and infected by injection of 10^6^ CFU of the CRE Nevada strain AR-0636 suspended in 100 μL PBS into the thigh muscle [80]. Mice were then injected with streptothricin-F administered subcutaneously at the scruff of the neck (i.e., distant from the thigh) 2 hours postinfection and euthanized after 24 hours. Thigh tissue was excised and homogenized using a disposable tissue grinder (Fisher Scientific) in 1 mL cation-adjusted Mueller–Hinton broth. Homogenates were serially diluted, plated on Mueller–Hinton agar plates (Remel, Lenexa, KS), and incubated at 37°C overnight for CFU enumeration. Animal research was approved by the Beth Israel Deaconess Medical Center Institutional Animal Care and Use Committee under protocol # #044–2015.

### Mutational resistance studies

Single ribosomal operon strain, SQ110, was grown overnight in CAMHB at 37°C and resuspended in one-fifth volume in fresh medium. Thereafter, 200 μL of the suspension was plated on selective LB agar plates containing nourseothricin (GoldBio, St Louis, MO) at 32-, 64-, and 128-fold the MIC of the parent SQ100 strain and incubated for 48 to 72 hours at 35°C in ambient air until mutant colonies became visible. Selected colonies were passaged on plates with matching concentrations of nourseothricin for further analysis. Aminoglycoside resistance mutants were isolated in a similar manner selecting on apramycin.

Genomic DNA from nourseothricin-resistant strains was extracted using the Wizard Genomic DNA Purification Kit (Promega, Madison, WI) from 3 mL of overnight culture in CAMHB containing nourseothricin (32 μg/mL, 64 μg/mL, and 128 μg/mL). Subsequent genomic DNA was amplified using primers F16S+23S, R16S+23S, and 16SR (S11 Table), with primer 16SR as previously described [72]. All amplification reactions were performed using Q5 high-fidelity DNA polymerase, Q5 reaction buffer, and DNTPs from New England Biolabs (Ipswich, MA) using a melting temperature of 68°C, an annealing temperature of 65°C, and an extension time of 2 minutes and 45 seconds. PCR products were purified using the QIAquick Purification Kit. Sanger sequencing was performed to identify mutations.

### Cryo-EM sample preparation

70S ribosomes were purified from *A*. *baumannii* (strain AB0057) using a sucrose cushion followed by sucrose gradient centrifugation [81] as we previously described [82]. Prior to cryo-EM grid preparation, ribosomes (0.1 μM) were incubated with 100 μM S-F or S-D for 2 hours. Cryo-EM samples were prepared by applying 3 μL of sample to graphene oxide-coated Quantifoil R1.2/1.3 grids, blotting for 15 seconds and plunge-freezing in liquid ethane using a Vitrobot (Thermo Fisher). Grids were then transferred into cartridges and stored in LN_2_ until data collection.

Cryo-EM data were recorded at a defocus range of −1 to −2.5 on a Titan Krios equipped with a K3 direct electron detector (Gatan). A total of 3,054 micrographs for S-F were recorded in superresolution mode at 105K magnification resulting in a physical pixel size of 0.848 Å (0.424 Å superresolution). Each image was collected over 40 frames with a total dose of 46 e/Å^2^ using SerialEM [83]. For S-D, 5,245 micrographs were recorded in superresolution mode at 81K magnification resulting in a physical pixel size of 1.08 Å (0.54 Å superresolution). Each image was collected over 40 frames with a total dose of 36 e/Å^2^ using SerialEM [83].

### Cryo-EM data processing

For both S-F and S-D, image stacks were aligned and binned by 0.5 (0.75 for S-D) using patch motion and the contrast transfer function (CTF) was estimated by patchCTF in cryoSPARC v3 [84]. A subset of micrographs was picked using blob picker to create initial templates for template picking, after which an initial pool of 648,714 particles for S-F and 1,749,230 particles were generated for S-D. Particles were cleaned using 2D classification and used to solve initial ab initio volumes. A modified Build-and-Retrieve (BaR) approach [85] using these initial maps were used to retrieve particles from the initial stack, which, followed by 2D and 3D cleaning, resulted in a notable increase in particle counts for each structure. 70S classes were further separated based off of tRNA population using three-dimensional variability analysis (3DVA) in cryosparc [84]. Refinements of the 3 structures for both S-F and S-D (70S P-site, 70S E-site, and Empty 70S) were split into 3 separate local refinements based off of motions in the 70S ribosome; the 50S, 30S core, and 30S head (S7 Fig). After an initial round of local refinement, final maps were refined after Local and Global CTF fitting and a final round of local refinement with nonuniform sampling. Final locally refined densities were post-processed in relion [86], and composite maps were created using vop maximum in Chimera [87].

### Model building and refinement

Previously determined structures of the *A*. *baumannii* ribosome [88] were used as starting models for the Ribosome-S-F and Ribosome-S-D complexes. Coot was first used to fit the initial model in the locally refined maps for each structure [89]. After initial modelling, only clear and complete densities found using the unmodeled blobs function in coot corresponding to S-F or S-D were modelled, resulting in S-F binding locations in the 30S head and 50S. In addition, only Mg^2+^ ions and coordinated waters that directly interact with S-F were included in the final model. Subsequent modelling and refinement were carried out in Coot [89] and phenix.real_space_refine from the PHENIX suite [90]. Final refinements were evaluated using MolProbity [91]. Final collection and refinement statistics are included in S7 and S8 Tables.

## Supporting information

S1 Results Spectral data for streptothricin F and streptothricin D; resistance studies provide complementary biological evidence that streptothricin and aminoglycoside targets are distinct. (PDF)

S1 Materials and Methods Characterization of purified streptothricin F and streptothricin D; cloning of 16S rRNA methylases and TetM; and assay of in vitro translation extracts prepared from 16S rRNA C1054A mutant ribosomes. (PDF)

S1 Table ^1^H NMR signals of streptothricin F and streptothricin D. (PDF)

S2 Table ^13^C NMR signals of streptothricin F and streptothricin D. (PDF)

S3 Table Streptothricin MIC data for individual CRE and *A*. *baumannii* strains. (PDF)

S4 Table The ratio of S-F to S-D minimal inhibitory concentration in *tolC* and *lptD* strain backgrounds was not significantly changed. (PDF)

S5 Table Genetic complementation of nourseothricin resistance mutants with wild-type *rrn* operon. (PDF)

S6 Table Effect of TetM on nourseothricin minimal inhibitory concentration in *Staphylococcus aureus* isolates. (PDF)

S7 Table Ribosome-S-F cryo-EM data collection and refinement statistics. (PDF)

S8 Table Ribosome-S-D cryo-EM data collection and refinement statistics. (PDF)

S9 Table Lack of effect of ribosomal 16S rRNA G1405 (ArmA) and A1408 (NpmA) methyltransferases on nourseothricin minimal inhibitory concentration (MIC in μg/mL). (PDF)

S10 Table 16s rRNA helix 34 and helix 44 mutations specifically inhibit streptothricin and aminoglycoside activity, respectively. (PDF)

S11 Table Primer sequences. (PDF)

S1 Fig Elemental analysis results of isolated streptothricin F from commercially available nourseothricin sulfate. (PDF)

S2 Fig Elemental analysis results of isolated streptothricin D from commercially available nourseothricin sulfate. (PDF)

S3 Fig Assignment of chemical shifts. The numbering scheme for streptothricin F and streptothricin D. In the ^1^H NMR, distinct chemical shifts for protons attached to the same carbon atom are labeled H_a_ and H_b_, respectively.
(PDF)

S4 Fig Rapid bactericidal activity of nourseothricin against the carbapenem-resistant *A*. *baumannii* isolate, MSRN1450. Nourseothricin MIC was 2 μg/mL.
(PDF)

S5 Fig Maps of vectors used in in vitro translational and ribosomal methylase inhibition analysis. (PDF)

S6 Fig Resistance of C1054A mutant ribosome to nourseothricin in in vitro translation assays. (PDF)

S7 Fig S-F cryo-EM processing workflow. (PDF)

S8 Fig S-D cryo-EM processing workflow. (PDF)

S9 Fig Final refined maps of *A*. *baumannii* 70S–S-F complexes. (PDF)

S10 Fig Final refined maps of *A*. *baumannii* 70S–S-D complexes. (PDF)

S11 Fig S-F binding sites in the *A*. *baumannii* 70S ribosome. (PDF)

S12 Fig S-D binding sites in the *A*. *baumannii* 70S ribosome. (PDF)

S13 Fig Similarities in the streptothricin binding site. (PDF)

S14 Fig Proposed S-F mechanism of action. (PDF)

S15 Fig TetM does not clash with S-F. (PDF)

S16 Fig Structural conservation of the S-F binding site in the human small ribosome subunit. (PDF)

S17 Fig Codon-optimized 16S rRNA methyltransferase resistance gene sequence. (PDF)

S1 Data Rapid bactericidal activity against the *Klebsiella pneumoniae* Nevada strain. Contains underlying data for Fig 2.
(XLSX)

S2 Data Rapid bactericidal activity of nourseothricin against the carbapenem-resistant *A*. *baumannii* isolate, MSRN1450. Contains underlying data for S4 Fig.
(XLSX)

S3 Data Inhibition of prokaryotic and eukaryotic translation. Contains underlying data for Fig 3.
(XLSX)

S4 Data Cytotoxicity for J774A.1 and LLK-PK-1 cells based on SYTOX-Green fluorescence assay. Contains underlying data for Fig 4.
(XLSX)

S5 Data Murine thigh infection model. Contains underlying data for Fig 6.
(XLSX)

S6 Data Resistance of C1054A mutant ribosome to nourseothricin in in vitro translation assays. Contains underlying data for S6 Fig.
(XLSX)

S7 Data GS-FSC curves of focus-refined regions of the 70S–S-F complex. Contains underlying data for S9C Fig.
(XLSX)

S8 Data GS-FSC curves of focus-refined regions of the 70S–S-D complex. Contains underlying data for S10C Fig.
(XLSX)

## Abbreviations

ALT: alanine aminotransferase
AST: aspartate aminotransferase
CRE: carbapenem-resistant Enterobacterales
cryo-EM: cryo-electron microscopy
IP: intraperitoneally
MIC: minimal inhibitory concentration
MTD: maximum tolerated dose
S-D: streptothricin D
S-F: streptothricin F
wt: wild-type; 2-DOS, 2-deoxystreptamine
