# Supplementary material for: Streptothricin F is a bactericidal antibiotic effective against highly drug-resistant gram-negative bacteria that interacts with the 30S subunit of the 70S ribosome
Source: PLoS Biol. 2023 May 16;21(5):e3002091. doi: 10.1371/journal.pbio.3002091 (PMC10187937; doi:10.1371/journal.pbio.3002091)
Supplement: S1 Results — (PDF) [file pbio.3002091.s001.pdf]

S1 Results

Spectral Data for Streptothricin F:

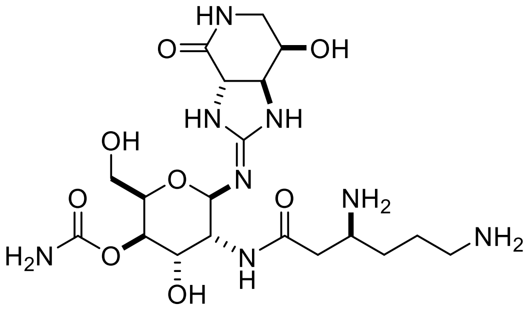

White solid: mp >210 °C. $[\alpha]_D^{21}$ = -43.45 (c = 0.1, H_2O). ^1H NMR (600 MHz, D_2O) δ 5.11 (d, J = 9.8 Hz, 1H), 4.76 (m, 1H), 4.76 (m, 1H), 4.63 (d, J = 13 Hz, 1H), 4.33 (t, J = 6.1 Hz, 1H), 4.24 (dd, J = 9.8, 2.8 Hz, 1H), 4.17 (t, J = 3.3 Hz, 1H), 4.08 (d, J = 14.3 Hz, 1H), 3.81 (dd, J = 14.7, 5.7 Hz, 1H), 3.75 – 3.65 (m, 3H), 3.39 (d, J = 14.6 Hz, 1H), 3.04 (t, J = 6.8 Hz, 2H), 2.81 (dd, J = 16.7, 4.3 Hz, 1H), 2.70 (dd, J = 16.6, 8.3 Hz, 1H), 1.80 (m, 4H). ^{13}C NMR (150 MHz, D_2O) δ 172.5, 56.82, 63.25, 62.72, 51.59, 165.14, 81.07, 51.46, 68.89, 72.43, 75.99, 62.62, 160.33, 174.51, 38.73, 50.65, 31.43, 25.25. HRMS-ESI (m / z): $[M + H]^+$ calcd for $C_{19}H_{35}N_8O_8$, 503.2577 Da; found 503.2574 Da. These results are consistent with the previously reported ^1H and ^{13}C NMR of streptothricin F from Ji et al (1).

Spectral Data for Streptothricin D:

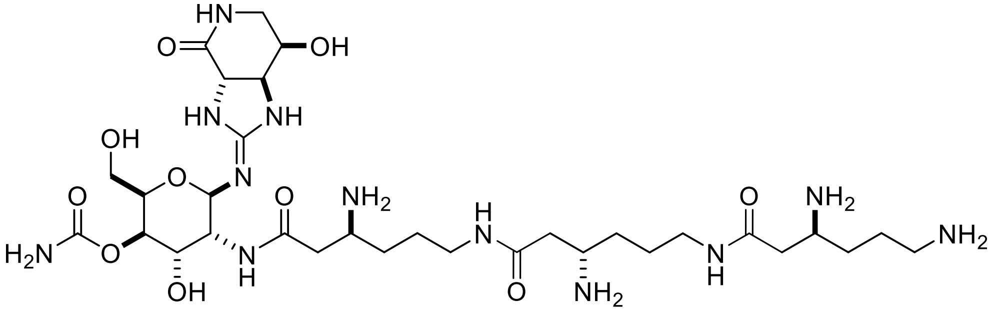

Faint yellow solid: mp >210 °C. $[\alpha]_D^{25}$ = -11.43 (c = 0.1, H_2O). ^1H NMR (600 MHz, D_2O) δ 5.14 (d, J = 9.8 Hz, 1H), 4.78 – 4.74 (m, 2H), 4.65 (d, J = 13.2, 1H), 4.35 (t, J = 6.2, 1H), 4.26 (dd, J = 3.0, 9.9 Hz, 1H), 4.18 (t, J = 3.4 Hz, 1H), 4.13 – 4.04 (m, 1H), 3.83 (dd, J = 5.7, 14.7 Hz, 1H), 3.78 – 3.61 (m, 6H), 3.42 (d, J = 14.7 Hz, 1H), 3.25 (t, J = 7.1 Hz, 4H), 3.07 (t, J = 6.8 Hz, 2H), 2.83 – 2.62 (m, 6H), 1.88 – 1.60 (m, 12H). ^{13}C NMR (150 MHz, D_2O) δ 172.62, 63.42, 63.42, 51.79, 165.27, 81.33, 51.60, 69.12, 72.62, 76.15, 62.90, 160.50, 174.80, 39.22, 51.06, 32.03, 26.82, 41.38, 174.51, 39.38, 51.05, 31.99, 26.78, 41.38, 174.35, 39.61, 51.27, 31.62, 25.42, 41.54. HRMS-ESI (m / z): $[M + H]^+$ calcd for $C_{31}H_{58}N_{12}O_{10}$, 759.4477 Da; found 759.4489 Da. These results are consistent with the previously reported ^1H and ^{13}C NMR of streptothricin D from Ji et al (1).

Elemental analysis was performed to confirm exact confirmation of streptothricin F (S-F) and streptothricin D (S-D) preparations and allow accurate conversion of weight to moles for determining relative biological activity in different experiments described in the main manuscript (S1 and S2 Figs) and Table S3. Results are consistent with the previously reported elemental analysis and molecular formula of S-D and S-F from Taniyama et al (2), respectively. S3 Fig provides the numbering scheme used for assignment of chemical shifts in NMR experiments.

Resistance studies provide complementary biological evidence that streptothricin and aminoglycoside targets are distinct. The antimicrobial activity of aminoglycosides is blocked by specific 16S rRNA methylases. Based on similarities between streptothricin and aminoglycoside activity, we considered the possibility that methylases that target the 16S rRNA A1408 position (e.g., NpmA) and block activity of all known 2-deoxystreptamine-based aminoglycosides including apramycin (3, 4), and methylases that target the 16S rRNA G1405 position (e.g., ArmA) and block activity of 4,6-disubstituted deoxystreptamine (DOS) aminoglycosides such as gentamicin, (but not apramycin), might interfere with activity of nourseothricin. Notably, in contrast to high-level resistance to gentamicin and apramycin controls, activity of nourseothricin (and by inference its component streptothricins) was completely unaffected by cloned *npmA* and *armA* methylases expressed from a pBAD promoter under inducing conditions (see S9 Table). Furthermore, the single ribosomal operon *E. coli* strain SQ110 (5) with either A1408G or G1491A mutations in 16S rRNA helix 44 that conferred high level resistance to aminoglycosides such as gentamicin, tobramycin, kanamycin, neomycin and apramycin did not affect nourseothricin susceptibility (S10 Table). These data also suggested that the target and corresponding mechanism of action of streptothricins are distinct from traditional aminoglycosides.

References

1. Ji Z, Wang M, Zhang J, Wei S, & Wu W (2007) Two new members of streptothricin class antibiotics from Streptomyces qinlingensis sp. nov. *J Antibiot (Tokyo)* 60(12):739-744.
2. Taniyama H, Sawada Y, & Kitagawa T (1971) Characterization of racemomycins. *Chem Pharm Bull* 19(8):1627-1634.
3. Doi Y, Wachino JI, & Arakawa Y (2016) Aminoglycoside resistance: The emergence of acquired 16S ribosomal RNA methyltransferases. *Infect Dis Clin North Am* 30(2):523-537.
4. Doi Y & Arakawa Y (2007) 16S Ribosomal RNA Methylation: Emerging Resistance Mechanism against Aminoglycosides. *Clinical Infectious Diseases* 45(1):88-94.
5. Orelle C*, et al.* (2013) Tools for characterizing bacterial protein synthesis inhibitors. *Antimicrob Agents Chemother* 57(12):5994-6004.
