# Supplementary material for: Streptothricin F is a bactericidal antibiotic effective against highly drug-resistant gram-negative bacteria that interacts with the 30S subunit of the 70S ribosome
Source: PLoS Biol. 2023 May 16;21(5):e3002091. doi: 10.1371/journal.pbio.3002091 (PMC10187937; doi:10.1371/journal.pbio.3002091)
Supplement: S1 Materials and Methods — (PDF) [file pbio.3002091.s002.pdf]

S1 Materials and Methods

Characterization of purified Streptothricin-F and Streptothricin-D. High-resolution mass spectra were performed on a LTQ Orbitrap XL via loop injection with an RSLC nano pump and were used to determine proportionality of streptothricins in the nourseothricin natural product used in experiments described in the main manuscript. NMR spectra were recorded at ambient temperature on a 600 MHz Bruker NMR spectrometer in D_2O. All ^1H NMR experiments are reported in δ units, parts per million (ppm) downfield of TMS, and were measured relative to the signals of water (4.79 ppm) with ^1H decoupled observation (S1 Table). ^{13}C NMR spectra were recorded with ^1H decoupled observation at ambient temperature on a Varian NMR spectrometer operating at 150 MHz in D_2O. Data for ^1H NMR are reported as follows: chemicals shift (δ ppm), multiplicity (s = singlet, d = doublet, dd = doublet of doublets, t = triplet, m = multiplet), integration, and coupling constant (Hz) whereas ^{13}C NMR analyses were reported in terms of chemical shift (S2 Table). NMR data was analyzed using MestReNova Software version 12.0.1. Polarimeter analysis was performed on a Jasco P-2000 Polarimeter using Spectra Manager 2.13.00 software.

Cloning of 16S rRNA methylases and TetM. The genes for the 16S rRNA G1405 and A1408 methylases, *armA* and *npmA*, were codon optimized for *E. coli* (S17 Fig) based using the ITD Codon Optimization Tool (https://www.idtdna.com/pages/tools/codon-optimization-tool), synthesized as a gBlock (IDT, Coralville, IA), and used to replace the LSSmOrange open reading frame in the arabinose-inducible vector, pBAD-LSSmOrange (1), a gift from Vladislav Verkhusha (Addgene plasmid # 37129; http://n2t.net/addgene:37129; RRID:Addgene_37129) by HiFi assembly (New England Biolabs, Beverely, MA) using primers, pBAD_F, pBAD_R, and armA_F

and arm A_R; and npmA F and npmA_R, respectively (Table S11) Assembled vectors (S5C and S5D Figs) were transformed into NEB 5-alpha (New England Biolabs), selecting for ampicillin and gentamicin resistance. TetM was amplified from *S. aureus* FDA-CDC strain 221 using primers tetM_F and tetM_R and cloned into pBAD-LSSmOrange, amplified using primers pBAD_F-tetM and pBAD_R-tetM using HiFi assembly as above. MIC assays were performed in the presence of 1% arabinose for resistance gene induction.

Assay of *in vitro* translation extracts prepared from 16S rRNA C1054A mutant ribosomes. SQ110 and C1054A (N1) strains were grown in 50 mL of LB medium overnight, centrifugated to pellet organisms and resuspended in 1 mL PBS with 1 mM dithiothreitol. The cell suspensions were lysed by sonication at 20 kHZ with a 3 mm diameter probe, at 35 % amplitude with 6 cycles of alternating 5-minute pulses and 1-minute rest for a total of 30 minutes sonication time (2). The lysates were centrifuged for 30 min at 4°C at 12000g, and supernatants were flash-frozen in liquid nitrogen and stored at -80 °C until further use. The cell-free *in vitro* transcription-translation reaction buffer was prepared and combined with bacterial extracts as described by Silverman et al. in their supplemental methods (3) with addition of 100 ng of plasmid pCR-XL-TOP-NLuc (see S5 Fig) to 30 µL reaction mixtures distributed in black 384-well microplates. Doubling dilutions of nourseothricin or apramycin were then added to wells in concentrations ranging from 0-200 µM using the TECAN D300 Digital Dispensing system. After incubation at 37°C for eighty minutes, 30 µL of PBS with 1 µg/mL furimazine (AOBIOUS, Gloucester, MA) was added to each well, and luminescence quantified using a TECAN M1000 microplate reader.

References

1. Shcherbakova DM, Hink MA, Joosen L, Gadella TW, & Verkhusha VV (2012) An orange fluorescent protein with a large Stokes shift for single-excitation multicolor FCCS and FRET imaging. *J Am Chem Soc* 134(18):7913-7923.
2. Shrestha P, Holland TM, & Bundy BC (2012) Streamlined extract preparation for Escherichia coli-based cell-free protein synthesis by sonication or bead vortex mixing. *Biotechniques* 53(3):163-174.
3. Silverman AD, Kelley-Loughnane N, Lucks JB, & Jewett MC (2019) Deconstructing Cell-Free Extract Preparation for in Vitro Activation of Transcriptional Genetic Circuitry. *ACS Synth Biol* 8(2):403-414.
