# Supplementary material for: Streptothricin F is a bactericidal antibiotic effective against highly drug-resistant gram-negative bacteria that interacts with the 30S subunit of the 70S ribosome
Source: PLoS Biol. 2023 May 16;21(5):e3002091. doi: 10.1371/journal.pbio.3002091 (PMC10187937; doi:10.1371/journal.pbio.3002091)
Supplement: S1 Table — (PDF) [file pbio.3002091.s003.pdf]

S1 Table. ^{1}H NMR signals of Streptothricin F and Streptothricin D.

Position	Isolated S-F, 600 MHz			Isolated S-D, 600 MHz		
	δ H (ppm)	mult	J (Hz)	δ H (ppm)	mult	J (Hz)
H-2	4.63	d	13	4.65	d	13.2
H-3	4.08	d	14.3	4.13-4.04	m	-
H-4	4.73	m	-	4.78-4.74	m	-
$H-5_a$	3.81	dd	5.7, 14.7	3.83	dd	5.7, 14.7
$H-5_b$	3.39	d	14.6	3.42	d	14.7
H-7	5.11	d	9.8	5.14	d	9.8
H-8	4.24	dd	2.8, 9.8	4.26	dd	3.0, 9.9
H-9	4.17	t	3.3	4.18	t	3.4
H-10	4.76	m	-	4.78-4.74	m	-
H-11	4.33	t	6.1	4.35	t	6.2
$H-12_a$	3.75-3.64	m	-	3.78-3.61	m	-
$H-12_b$	3.75-3.64	m	-	3.78-3.61	m	-
$H-15_a$	2.81	dd	4.3, 16.7	2.83-2.62	m	-
$H-15_b$	2.7	dd	8.3, 16.6	2.83-2.62	m	-
H-16	3.75-3.64	m	-	3.78-3.61	m	-
H-17	1.8	m		1.86-1.58	m	-
H-18	1.8	m		1.86-1.58	m	-
H-19	3.04	t	6.8	3.25	t	7.1
$H-21_a$	-	-	-	2.83-2.62	m	-
$H-21_b$	-	-	-	2.83-2.62	m	-
H-22	-	-	-	3.78-3.61	m	-
H-23	-	-	-	1.86-1.58	m	-
H-24	-	-	-	1.86-1.58	m	-
H-25	-	-	-	3.25	t	7.1
$H-27_a$	-	-	-	2.83-2.62	m	-
$H-27_b$	-	-	-	2.83-2.62	m	-
H-28	-	-	-	3.78-3.61	m	-
H-29	-	-	-	1.86-1.58	m	-
H-30	-	-	-	1.86-1.58	m	-
H-31	-	-	-	3.07	t	6.8
