# Supplementary material for: Streptothricin F is a bactericidal antibiotic effective against highly drug-resistant gram-negative bacteria that interacts with the 30S subunit of the 70S ribosome
Source: PLoS Biol. 2023 May 16;21(5):e3002091. doi: 10.1371/journal.pbio.3002091 (PMC10187937; doi:10.1371/journal.pbio.3002091)
Supplement: S2 Table — (PDF) [file pbio.3002091.s004.pdf]

S2 Table. ^{13}C NMR signals of Streptothricin F and Streptothricin D.

Position	Isolated Streptothricin F, 600 MHz δ (ppm)	Isolated Streptothricin D, 600 MHz δ H (ppm)
1	172.5	172.62
2	56.82	56.98 (broad)
3	63.25	63.42
4	62.72	63.42
5	51.59	51.79
6	165.14	165.27
7	81.07	81.33
8	51.46	51.60
9	68.89	69.12
10	72.43	72.62
11	75.99	76.15
12	62.62	62.90
13	160.33	160.50
14	174.51	174.80
15	38.73	39.22
16	50.65	51.06
17	31.43	32.03
18	25.25	26.82
19	41.35	41.38
20	-	174.51
21	-	39.38
22	-	51.05
23	-	31.99
24	-	26.78
25	-	41.38
26	-	174.35
27	-	39.61
28	-	51.27
29	-	31.62
30	-	25.42
31	-	41.54
