# Supplementary material for: Streptothricin F is a bactericidal antibiotic effective against highly drug-resistant gram-negative bacteria that interacts with the 30S subunit of the 70S ribosome
Source: PLoS Biol. 2023 May 16;21(5):e3002091. doi: 10.1371/journal.pbio.3002091 (PMC10187937; doi:10.1371/journal.pbio.3002091)
Supplement: S3 Table — (PDF) [file pbio.3002091.s005.pdf]

S3 Table. Streptothricin MIC data for individual CRE and *A. baumannii* strains.

Collection	Number	Genus	Species	Resistance Element[a]	S-D MIC (μM)	S-F MIC (μM)	Nour MIC (μM)
BIDMC	1	*Klebsiella*	*pneumoniae*	KPC-3	0.25	2	0.5
BIDMC	2	*Klebsiella*	*pneumoniae*	KPC-3	0.25	4	0.5
BIDMC	3	*Escherichia*	*coli*	KPC-2	0.5	4	0.5
FDA-CDC	34	*Klebsiella*	*pneumoniae*	IMP-4	0.25	2	0.25
FDA-CDC	117	*Klebsiella*	*pneumoniae*	KPC-3	0.25	1	0.25
FDA-CDC	118	*Escherichia*	*coli*	NDM	1	4	2
FDA-CDC	119	*Escherichia*	*coli*	NDM	0.5	4	1
FDA-CDC	128	*Escherichia*	*coli*	NDM-1	1	4	1
FDA-CDC	161	*Enterobacter*	*aerogenes*	IMP-4	0.5	2	1
CDC	636	*Klebsiella*	*pneumoniae*	NDM	0.25	1	0.25
BIDMC	4	*Klebsiella*	*pneumoniae*	KPC-3	0.25	2	0.25
BIDMC	5	*Klebsiella*	*pneumoniae*	KPC-3	0.25	2	0.25
BIDMC	6	*Escherichia*	*coli*	KPC-2	0.5	2	0.5
BIDMC	7	*Klebsiella*	*pneumoniae*	KPC-3	0.25	2	0.5
BIDMC	8	*Enterobacter*	*cloacae*	KPC-3	0.25	4	0.5
BIDMC	9	*Escherichia*	*coli*	KPC-2	0.5	4	0.5
BIDMC	10	*Klebsiella*	*pneumoniae*	KPC-3	0.25	2	0.5
BIDMC	11	*Klebsiella*	*pneumoniae*	KPC-4	0.25	2	0.25
BIDMC	12	*Klebsiella*	*pneumoniae*	KPC-3	0.5	2	0.5
BIDMC	13	*Klebsiella*	*pneumoniae*	KPC-3	0.25	2	0.5
BIDMC	14	*Klebsiella*	*pneumoniae*	KPC-3	0.5	2	0.5
BIDMC	15	*Escherichia*	*coli*	KPC-2	0.5	2	0.5
BIDMC	16	*Klebsiella*	*pneumoniae*	KPC-3	0.25	2	0.5
BIDMC	17	*Escherichia*	*coli*	KPC-2	0.5	2	0.5
BIDMC	18	*Klebsiella*	*pneumoniae*	KPC-2	2	4	2
FDA-CDC	135	*Klebsiella*	*pneumoniae*	VIM	0.5	2	0.5
FDA-CDC	136	*Enterobacter*	*cloacae*	KPC	0.5	2	0.5
FDA-CDC	137	*Escherichia*	*coli*	NDM-6	0.5	2	1
FDA-CDC	138	*Klebsiella*	*pneumoniae*	NDM	0.25	1	0.25
FDA-CDC	139	*Klebsiella*	*pneumoniae*	NDM-1	0.25	2	0.5
FDA-CDC	140	*Klebsiella*	*pneumoniae*	OXA-181	0.25	2	0.5
FDA-CDC	141	*Klebsiella*	*pneumoniae*	OXA-181	0.5	4	0.5

FDA-CDC	142	*Klebsiella*	*pneumoniae*	OXA-181	0.25	2	0.25
FDA-CDC	143	*Klebsiella*	*pneumoniae*	NDM-1	0.25	2	0.5
FDA-CDC	145	*Klebsiella*	*pneumoniae*	NDM	0.5	2	0.5
FDA-CDC	147	*Klebsiella*	*oxytoca*	KPC	0.25	2	0.5
FDA-CDC	148	*Klebsiella*	*pneumoniae*	NDM	0.25	2	0.5
FDA-CDC	149	*Escherichia*	*coli*	NDM	0.5	4	0.5
FDA-CDC	150	*Escherichia*	*coli*	NDM-5	0.5	2	0.5
FDA-CDC	308	*Acinetobacter*	*baumannii*	OXA-71	0.25	1	0.25
FDA-CDC	309	*Acinetobacter*	*baumannii*	OXA-23<br>OXA-82	2	8	2
FDA-CDC	310	*Acinetobacter*	*baumannii*	OXA-23<br>OXA-82	2	8	2
FDA-CDC	311	*Acinetobacter*	*baumannii*	OXA-23<br>OXA-82	2	8	2
FDA-CDC	33	*Acinetobacter*	*baumannii*		nt	nt	0.5
FDA-CDC	35	*Acinetobacter*	*baumannii*		nt	nt	8
FDA-CDC	36	*Acinetobacter*	*baumannii*		nt	nt	16
FDA-CDC	37	*Acinetobacter*	*baumannii*		nt	nt	0.5
FDA-CDC	45	*Acinetobacter*	*baumannii*		nt	nt	2
FDA-CDC	52	*Acinetobacter*	*baumannii*		nt	nt	0.5
FDA-CDC	56	*Acinetobacter*	*baumannii*		nt	nt	2
FDA-CDC	63	*Acinetobacter*	*baumannii*		nt	nt	2
FDA-CDC	70	*Acinetobacter*	*baumannii*		nt	nt	0.5
FDA-CDC	78	*Acinetobacter*	*baumannii*		nt	nt	4
FDA-CDC	83	*Acinetobacter*	*baumannii*		nt	nt	4
FDA-CDC	88	*Acinetobacter*	*baumannii*		nt	nt	2
FDA-CDC	101	*Acinetobacter*	*baumannii*		nt	nt	8
FDA-CDC	102	*Acinetobacter*	*baumannii*		nt	nt	2
FDA-CDC	0273	*Acinetobacter*	*baumannii*	CRAB	nt	nt	2
FDA-CDC	0274	*Acinetobacter*	*baumannii*	CRAB	nt	nt	16
FDA-CDC	0275	*Acinetobacter*	*baumannii*	CRAB	nt	nt	2
FDA-CDC	0276	*Acinetobacter*	*baumannii*	CRAB	nt	nt	64
FDA-CDC	0277	*Acinetobacter*	*baumannii*	CRAB	nt	nt	8
FDA-CDC	0278	*Acinetobacter*	*baumannii*	CRAB	nt	nt	4
FDA-CDC	0279	*Acinetobacter*	*baumannii*	CRAB	nt	nt	4
FDA-CDC	0280	*Acinetobacter*	*baumannii*	CRAB	nt	nt	4

FDA-CDC	0281	*Acinetobacter*	*baumannii*	CRAB	nt	nt	4
FDA-CDC	0282	*Acinetobacter*	*baumannii*	CRAB	nt	nt	4
FDA-CDC	0283	*Acinetobacter*	*baumannii*	CRAB	nt	nt	4
FDA-CDC	0284	*Acinetobacter*	*baumannii*	CRAB	nt	nt	8
FDA-CDC	0285	*Acinetobacter*	*baumannii*	CRAB	nt	nt	4
FDA-CDC	0286	*Acinetobacter*	*baumannii*	CRAB	nt	nt	8
FDA-CDC	0287	*Acinetobacter*	*baumannii*	CRAB	nt	nt	2
FDA-CDC	0288	*Acinetobacter*	*baumannii*	CRAB	nt	nt	2
FDA-CDC	0289	*Acinetobacter*	*baumannii*	CRAB	nt	nt	2
FDA-CDC	0290	*Acinetobacter*	*baumannii*	CRAB	nt	nt	4
FDA-CDC	0291	*Acinetobacter*	*baumannii*	CRAB	nt	nt	2
FDA-CDC	0292	*Acinetobacter*	*baumannii*	CRAB	nt	nt	2
FDA-CDC	0293	*Acinetobacter*	*baumannii*	CRAB	nt	nt	2
FDA-CDC	0294	*Acinetobacter*	*baumannii*	CRAB	nt	nt	2
FDA-CDC	0295	*Acinetobacter*	*baumannii*	CRAB	nt	nt	4
FDA-CDC	0296	*Acinetobacter*	*baumannii*	CRAB	nt	nt	4
FDA-CDC	0297	*Acinetobacter*	*baumannii*	CRAB	nt	nt	32
FDA-CDC	0298	*Acinetobacter*	*baumannii*	CRAB	nt	nt	2
FDA-CDC	0299	*Acinetobacter*	*baumannii*	CRAB	nt	nt	8
FDA-CDC	0300	*Acinetobacter*	*baumannii*	CRAB	nt	nt	4
FDA-CDC	0301	*Acinetobacter*	*baumannii*	CRAB	nt	nt	8
FDA-CDC	0302	*Acinetobacter*	*baumannii*	CRAB	nt	nt	4
FDA-CDC	0303	*Acinetobacter*	*baumannii*	CRAB	nt	nt	2
FDA-CDC	0304	*Acinetobacter*	*baumannii*	CRAB	nt	nt	4
FDA-CDC	0305	*Acinetobacter*	*baumannii*	CRAB	nt	nt	8
FDA-CDC	0306	*Acinetobacter*	*baumannii*	CRAB	nt	nt	8
FDA-CDC	0307	*Acinetobacter*	*baumannii*	CRAB	nt	nt	4
FDA-CDC	312	*Acinetobacter*	*baumannii*	CRAB	nt	nt	1
MRSN	7117	*Acinetobacter*	*baumannii*		nt	nt	2
MRSN	7322	*Acinetobacter*	*baumannii*		nt	nt	2
MRSN	7436	*Acinetobacter*	*baumannii*		nt	nt	4
MRSN	7465	*Acinetobacter*	*baumannii*		nt	nt	0.25
MRSN	7520	*Acinetobacter*	*baumannii*		nt	nt	0.5
MRSN	11684	*Acinetobacter*	*baumannii*		nt	nt	2
MRSN	11712	*Acinetobacter*	*baumannii*		nt	nt	0.5
MRSN	11751	*Acinetobacter*	*baumannii*		nt	nt	0.25
MRSN	15038	*Acinetobacter*	*baumannii*		nt	nt	<0.125
MRSN	15067	*Acinetobacter*	*baumannii*		nt	nt	0.25
MRSN	55	*Acinetobacter*	*baumannii*	CRAB	nt	nt	0.25

MRSN	846	*Acinetobacter*	*baumannii*	CRAB	nt	nt	0.5
MRSN	847	*Acinetobacter*	*baumannii*	CRAB	nt	nt	0.5
MRSN	865	*Acinetobacter*	*baumannii*	CRAB	nt	nt	2
MRSN	881	*Acinetobacter*	*baumannii*	CRAB	nt	nt	0.5
MRSN	890	*Acinetobacter*	*baumannii*	CRAB	nt	nt	0.25
MRSN	899	*Acinetobacter*	*baumannii*	CRAB	nt	nt	1
MRSN	915	*Acinetobacter*	*baumannii*	CRAB	nt	nt	0.5
MRSN	1424	*Acinetobacter*	*baumannii*	CRAB	nt	nt	2
MRSN	1450	*Acinetobacter*	*baumannii*	CRAB	nt	nt	2
MRSN	1625	*Acinetobacter*	*baumannii*	CRAB	nt	nt	4
MRSN	2001	*Acinetobacter*	*baumannii*	CRAB	nt	nt	1
MRSN	2356	*Acinetobacter*	*baumannii*	CRAB	nt	nt	0.25
MRSN	2400	*Acinetobacter*	*baumannii*	CRAB	nt	nt	8
MRSN	2402	*Acinetobacter*	*baumannii*	CRAB	nt	nt	1
MRSN	3360	*Acinetobacter*	*baumannii*	CRAB	nt	nt	2
MRSN	3384	*Acinetobacter*	*baumannii*	CRAB	nt	nt	2
MRSN	3607	*Acinetobacter*	*baumannii*	CRAB	nt	nt	2
MRSN	3716	*Acinetobacter*	*baumannii*	CRAB	nt	nt	4
MRSN	7271	*Acinetobacter*	*baumannii*	CRAB	nt	nt	2
MRSN	7339	*Acinetobacter*	*baumannii*	CRAB	nt	nt	4
MRSN	7401	*Acinetobacter*	*baumannii*	CRAB	nt	nt	0.5
MRSN	7493	*Acinetobacter*	*baumannii*	CRAB	nt	nt	4
MRSN	11656	*Acinetobacter*	*baumannii*	CRAB	nt	nt	2
MRSN	11663	*Acinetobacter*	*baumannii*	CRAB	nt	nt	2
MRSN	11672	*Acinetobacter*	*baumannii*	CRAB	nt	nt	4
MRSN	11681	*Acinetobacter*	*baumannii*	CRAB	nt	nt	32
MRSN	11693	*Acinetobacter*	*baumannii*	CRAB	nt	nt	32
MRSN	11755	*Acinetobacter*	*baumannii*	CRAB	nt	nt	32
MRSN	11777	*Acinetobacter*	*baumannii*	CRAB	nt	nt	32
MRSN	13300	*Acinetobacter*	*baumannii*	CRAB	nt	nt	32
MRSN	15081	*Acinetobacter*	*baumannii*	CRAB	nt	nt	32
MRSN	17480	*Acinetobacter*	*baumannii*	CRAB	nt	nt	32
MRSN	17990	*Acinetobacter*	*baumannii*	CRAB	nt	nt	32
MRSN	19209	*Acinetobacter*	*baumannii*	CRAB	nt	nt	32
MRSN	22623	*Acinetobacter*	*baumannii*	CRAB	nt	nt	4
MRSN	23016	*Acinetobacter*	*baumannii*	CRAB	nt	nt	1
MRSN	24384	*Acinetobacter*	*baumannii*	CRAB	nt	nt	2
MRSN	28691	*Acinetobacter*	*baumannii*	CRAB	nt	nt	0.5
MRSN	30728	*Acinetobacter*	*baumannii*	CRAB	nt	nt	1

[a]β-lactamase presumptively associated with carbapenem resistance phenotype.
Nour = nourseothricin; BIDMC = Beth Israel Deaconess Medical Center; FDA-CDC = Antimicrobial Resistance Isolate Bank Collection; MSRN=Multidrug Resistant Organism Repository and Surveillance Network (MRSN) at the Walter Reed Army Institute of Research; CRAB = carbapenem-resistant *A. baumannii*; nt = not tested
