# Supplementary material for: Streptothricin F is a bactericidal antibiotic effective against highly drug-resistant gram-negative bacteria that interacts with the 30S subunit of the 70S ribosome
Source: PLoS Biol. 2023 May 16;21(5):e3002091. doi: 10.1371/journal.pbio.3002091 (PMC10187937; doi:10.1371/journal.pbio.3002091)
Supplement: S4 Table — (PDF) [file pbio.3002091.s006.pdf]

S4 Table. The ratio of S-F to S-D minimal inhibitory concentration in *tolC* and *lptD* strain background was not significantly changed.

experiment 1	**wt**	***tolC***	***lptD***
**S-F**	2	2	1
**S-D**	0.5	0.25	0.125
**S-F:S-D ratio**	4	8	8

experiment 2	**wt**	***tolC***	***lptD***
**S-F**	4	2	0.5
**S-D**	0.5	0.25	0.0625
**S-F:S-D ratio**	8	8	8

Modal MIC in µM values for three (experiment 1) or four technical replicates (experiment 2). The similarity of ratios between S-F and S-D suggested that there was not a significant difference in either efflux (*tolC*) or permeability (*lptD*) between these two streptothricins.by these measures. Abbreviations: **wt** = *E. coli* strain BW25113 from the Coli Genetics Stock Center (CGSC, Yale University) # 7636; ***tolC*** = *E. coli* strain JW5503-KanS, CGSC # 14206; ***lptD*** = *E. coli* strain RFM795, CGSC # 14179.
