# Supplementary material for: Streptothricin F is a bactericidal antibiotic effective against highly drug-resistant gram-negative bacteria that interacts with the 30S subunit of the 70S ribosome
Source: PLoS Biol. 2023 May 16;21(5):e3002091. doi: 10.1371/journal.pbio.3002091 (PMC10187937; doi:10.1371/journal.pbio.3002091)
Supplement: S5 Table — (PDF) [file pbio.3002091.s007.pdf]

S5 Table. Genetic complementation of nourseothricin resistance mutants with wild type *rrn* operon.

**strain**		**SQ110**	**N1**	**N8**	**N9**	**N11**	**N12**	**N13**
**genotype**		**wt**	**C1054A**	**C1054A**	**A1196G**	**A1196G**	**A1196C**	**A1196C**
**nourseothricin**	control	0.12	≥256	≥256	8	8	8	8
**nourseothricin**	*rrnB* vector	1	0.25	0.5	1	0.5	1	2
**gentamicin**	control	0.5	0.5	1	1	1	1	1
**gentamicin**	*rrnB* vector	0.06	≤0.03	≤0.03	0.06	0.06	0.06	0.06

Wild type SQ110 and mutant strains were transformed with pKK3535 expressing the wild type *rrnB* operon (expressing ribosomal RNA genes) from *E. coli* K-12 (labeled *rrnB* vector) or its pBR322 vector backbone (labeled control). Activity of nourseothricin and gentamicin was assessed by MIC analysis. The wild type ribosomal operon completely restored susceptibility in nourseothricin resistant mutants consistent with linkage of the specific mutations (C1054A, A1196G, and A1196C) with the resistance phenotype and the recessive nature of the resistance mutations. Two separately isolated mutants of each type were tested as indicated by individual strain names. Data represent the modal value for three to six technical replicates. pKK3535 was isolated from SQ171 obtained from the Coli Genetics Stock Center, strain # 12384.
