# Supplementary material for: Streptothricin F is a bactericidal antibiotic effective against highly drug-resistant gram-negative bacteria that interacts with the 30S subunit of the 70S ribosome
Source: PLoS Biol. 2023 May 16;21(5):e3002091. doi: 10.1371/journal.pbio.3002091 (PMC10187937; doi:10.1371/journal.pbio.3002091)
Supplement: S6 Table — (PDF) [file pbio.3002091.s008.pdf]

S6 Table. Effect of TetM on nourseothricin minimal inhibitory concentration in *Staphylococcus aureus* isolates.

	strains		
	**ATCC 25923**	**AR-219**	**AR-221**
**TetM**	**-**	+	+
**nourseothricin**	0.5	1	1
**tetracycline**	0.5	32	64

Minimal inhibitory concentrations in µg/mL represent the modal value for three biological replicates. The presence (+) or absence (-) of the TetM ribosomal protection protein gene are as indicated. No other specific tetracycline resistance elements were identified in AR-219 (Genbank ID: CP029675.1, CP029674.1), AR-221 (Genbank ID: NZ_QHCN01000001.1, NZ_QHCN01000002.1, NZ_QHCN01000003.1), and ATCC 25923 (Genbank ID: NZ_CP009361) in a CARD resistance gene identifier (RGI 5.2.0, CARD 3.1.2) search (1) performed May 24, 2021 using perfect and strict hit settings.

Reference

1. Alcock BP, *et al.* (2020) CARD 2020: antibiotic resistome surveillance with the comprehensive antibiotic resistance database. *Nucleic Acids Res* 48(D1):D517-d525.
