# Supplementary material for: Streptothricin F is a bactericidal antibiotic effective against highly drug-resistant gram-negative bacteria that interacts with the 30S subunit of the 70S ribosome
Source: PLoS Biol. 2023 May 16;21(5):e3002091. doi: 10.1371/journal.pbio.3002091 (PMC10187937; doi:10.1371/journal.pbio.3002091)
Supplement: S7 Table — (PDF) [file pbio.3002091.s009.pdf]

S7 Table. Ribosome-S-F cryo-EM data collection and refinement statistics.

**Data collection**			
Magnification	105,000		
Voltage (kV)	300		
Electron Microscope	Krios-GIF-K3		
Defocus (um)	-1.0 to -2.5		
Energy filter width (eV)	20		
Pixel size (Å)[a]	0.848 (0.424)		
Total dose (e^{-}/ $Å^2$)	46		
Number of frames	40		
Number of micrographs	3,054		
Initial particle images (no.)	648,714		
Final particle images (no.)	430,291		
**Refinement**	**70S P-site-tRNA**	**70S E-site-tRNA**	**70S Empty**
Total Particles (no.)	53,114	109,944	135,858
GS-FSC Resolution (0.143, Å)[b]			
50S	2.50	2.37	2.35
30S Core	2.72	2.59	2.55
30S Head	2.66	2.52	2.57
Model composition			
Chains	53	53	51
Protein residues	5,500	5,512	5,510
Nucleotides	4,447	4,442	4,358
Streptothricin-F	2	0	1
r.m.s.d.			
Bond lengths (Å)	0.004	0.004	0.003
Bond angles (°)	0.622	0.621	0.588
**Validation**	**70S P-site-tRNA**	**70S E-site-tRNA**	**70S Empty**
MolProbity score	1.72	1.66	1.64
Clash score	5.07	4.71	4.45
Ramachandran plot			
Favored (%)	97.22	97.23	97.47
Allowed (%)	2.63	2.64	2.33
Disallowed (%)	0.15	0.13	0.20
CC Mask	0.91	0.91	0.91

[a]Super-resolution stacks processed in 0.75 resolution instead of 0.5 during motion correction
[b]Gold-Standard Fourier Shell Correlation; refinements split into three regions for each structure.
