# Supplementary material for: Streptothricin F is a bactericidal antibiotic effective against highly drug-resistant gram-negative bacteria that interacts with the 30S subunit of the 70S ribosome
Source: PLoS Biol. 2023 May 16;21(5):e3002091. doi: 10.1371/journal.pbio.3002091 (PMC10187937; doi:10.1371/journal.pbio.3002091)
Supplement: S8 Table — (PDF) [file pbio.3002091.s010.pdf]

S8 Table. Ribosome-S-D cryo-EM data collection and refinement statistics.

**Data collection**			
Magnification		80,000	
Voltage (kV)		300	
Electron Microscope		Krios-GIF-K3	
Defocus (um)		-1.0 to -2.5	
Energy filter width (eV)		20	
Pixel size (Å)[a]		1.08 (0.54)	
Total dose (e^-/ $Å^2$)		36	
Number of frames		40	
Number of micrographs		5,245	
Initial particle images (no.)		1,749,230	
Final particle images (no.)		1,251,104	
**Refinement**	**70S P-site-tRNA**	**70S E-site-tRNA**	**70S Empty**
Total Particles (no.)	144,189	451,971	420,107
GS-FSC Resolution (0.143, Å)[b]			
50S	2.39	2.21	2.21
30S Core	2.67	2.45	2.47
30S Head	2.65	2.52	2.51
Model composition			
Chains	53	53	51
Protein residues	5,512	5,512	5,510
Nucleotides	4,447	4,453	4,369
Streptothricin-D	12	11	12
r.m.s.d.			
Bond lengths (Å)	0.005	0.005	0.004
Bond angles (°)	0.663	0.615	0.604
**Validation**	**70S P-site-tRNA**	**70S E-site-tRNA**	**70S Empty**
MolProbity score	1.69	1.55	1.49
Clash score	4.90	4.61	4.26
Ramachandran plot			
Favored (%)	97.25	97.69	97.67
Allowed (%)	2.55	2.14	2.14
Disallowed (%)	0.20	0.17	0.18
CC Mask	0.93	0.93	0.93

[a]Super-resolution stacks processed in 0.75 resolution instead of 0.5 during motion correction
[b]Gold-Standard Fourier Shell Correlation; refinements split into three regions for each structure.
