# Supplementary material for: Streptothricin F is a bactericidal antibiotic effective against highly drug-resistant gram-negative bacteria that interacts with the 30S subunit of the 70S ribosome
Source: PLoS Biol. 2023 May 16;21(5):e3002091. doi: 10.1371/journal.pbio.3002091 (PMC10187937; doi:10.1371/journal.pbio.3002091)
Supplement: S9 Table — (PDF) [file pbio.3002091.s011.pdf]

S9 Table. Lack of effect of ribosomal 16S rRNA G1405 (ArmA) and A1408 (NpmA) methyltransferases on nourseothricin minimal inhibitory concentration (MIC in µg/mL).

Antibiotic	*armA*[a]	*npmA*[a]	vector control[b]
gentamicin	>256	16	0.125
apramycin	1	> 256	1
nourseothricin	0.25	0.25	0.5

[a]genes cloned under inducible pBAD promoter (see S5 Fig). 1% arabinose added to MIC panels for induction.

[b]Vector control was the same pBAD vector expressing an unrelated protein (LSSOrange) cloned in the same position as the *armA* and *npmA* methyltransferases, providing a measure of the susceptibility of the strains in the absence of any resistance gene.

MIC values represent mode for at least 8 separate determinations in two biological replicates.
