# Supplementary material for: Streptothricin F is a bactericidal antibiotic effective against highly drug-resistant gram-negative bacteria that interacts with the 30S subunit of the 70S ribosome
Source: PLoS Biol. 2023 May 16;21(5):e3002091. doi: 10.1371/journal.pbio.3002091 (PMC10187937; doi:10.1371/journal.pbio.3002091)
Supplement: S10 Table — (PDF) [file pbio.3002091.s012.pdf]

**S10 Table. 16s rRNA helix 34 and helix 44 mutations specifically inhibit streptothricin and aminoglycoside activity, respectively.**

| | Mutation | NAT | S-F | GEN | TOB | KAN | NEO | APR | PARO | RIB |
|---|---|---|---|---|---|---|---|---|---|---|
| | wt | 1 | 2 | 0.25 | 0.5 | 2 | 0.5 | 16 | 2 | 2 |
| helix 34 | C1054A | 512 | >512 | 0.25 | 0.25 | 1 | 0.25 | 1 | 0.5 | 1 |
| | C1054A | 512 | >512 | 0.25 | 0.25 | 0.5 | 0.25 | 1 | 0.25 | 0.5 |
| helix 44 | A1408G | 1 | 2 | 64 | 512 | >512 | 512 | >512 | 32 | >512 |
| | A1408G | 0.5 | 2 | 32 | 512 | >512 | 512 | >512 | 16 | >512 |
| | A1408G | 1 | 2 | 32 | 512 | >512 | 512 | >512 | 16 | >512 |
| | G1491A | 0.5 | 1 | 0.5 | 2 | 8 | 1 | 512 | 16 | 128 |
| | G1491A | 0.5 | 2 | 0.5 | 2 | 4 | 1 | 512 | 16 | 128 |
| | G1491A | 0.5 | 2 | 0.5 | 2 | 8 | 2 | >512 | 32 | 256 |

Minimal inhibitory concentration results for individually isolated aminoglycoside mutants in the single ribosomal operon SQ110 strain (wt) isolated on 128 µg/mL apramycin (helix 44 mutants) or nourseothricin (helix 34 mutants) as described in Materials and Methods. Aminoglycoside MIC values are in µg/mL; streptothricins, in µM. NAT = nourseothricin; S-F = streptothricin F; GEN = gentamicin; TOB = tobramycin; KAN = kanamycin; NEO = neomycin; APR = apramycin; PARO = paromomycin; RIB = ribostamycin.
