# Supplementary material for: Streptothricin F is a bactericidal antibiotic effective against highly drug-resistant gram-negative bacteria that interacts with the 30S subunit of the 70S ribosome
Source: PLoS Biol. 2023 May 16;21(5):e3002091. doi: 10.1371/journal.pbio.3002091 (PMC10187937; doi:10.1371/journal.pbio.3002091)
Supplement: S11 Table — (PDF) [file pbio.3002091.s013.pdf]

S11 Table. Primer Sequences.

Primer Name	Sequence
pBAD_F	GAATTCGAAGCTTGGCTGT
pBAD_R	GGTTAATTCCTCCTGTTAGCCC
ArmA_F	GGCTAACAGGAGGAATTAACCATGGA CAAGAATGACGTGGTCAAAAAA
ArmA_R	ACAGCCAAGCTTCGAATTCTTACTTTTGGAAGCCCGAAGT
NpmA_F	GGCTAACAGGAGGAATTAACCATGCT GATTCTGAAAGGAACTAAAACGG
NpmA_R	ACAGCCAAGCTTCGAATTCTTAGTGTTTCGATACATGACCGG
T7Nluc-F	GACGTTGTAAAACGA
pBAD-tetM_F	TGTTCAATAAAATAACTTAGgaattcgaagcttggctgttttgg
pBAD tetM_R	CCAATATTAATAATTTTCATggttaattcctcctgttagccC
tetM_F	gctaacaggaggaattaaccATGAAAATTATTAATATTGGAGTTTTAGCTCAT GTTGATGCAGG
tetM_R	aacagccaagcttcgaattcCTAAGTTATTTTATTGAACATATATCTTACTTTA TCTATCCGACT
T7Nluc-R	AGGAAGCCCGGATAT
NLuc_BamHI-F	CGC *GGATCC* GTCTTCACACTCGAAGAT
NLuc_PstI-R	CGC *CTGCAG* TTACGCCAGAATGCGTTC
F16S+23S	CACCTCGCGACAGTGCGCTAAAGCG
R16S+23S	GGTCAGGTGGGACCACCGCGCTAC
16SR*	CGCAAGACGCCTTGCTTTTCA

*16S rRNA *rrsE* reverse primer from Orelle C, et al. (1).

Reference

1. Orelle C*, et al.* (2013) Tools for characterizing bacterial protein synthesis inhibitors. *Antimicrob Agents Chemother* 57(12):5994-6004.
