# Supplementary material for: Streptothricin F is a bactericidal antibiotic effective against highly drug-resistant gram-negative bacteria that interacts with the 30S subunit of the 70S ribosome
Source: PLoS Biol. 2023 May 16;21(5):e3002091. doi: 10.1371/journal.pbio.3002091 (PMC10187937; doi:10.1371/journal.pbio.3002091)
Supplement: S1 Fig — (PDF) [file pbio.3002091.s014.pdf]

S1 Fig. Elemental analysis results of isolated streptothricin F from commercially available nourseothricin sulfate. The percent compositions align with a molecular formula of $C_{19}H_{34}N_8O_8$ • 3/2 H_2SO_4 • 3 H_2O.

C	H	N	O
32.47 %	6.48 %	14.01 %	36.68 %

S
7.12 %
