# Supplementary material for: Streptothricin F is a bactericidal antibiotic effective against highly drug-resistant gram-negative bacteria that interacts with the 30S subunit of the 70S ribosome
Source: PLoS Biol. 2023 May 16;21(5):e3002091. doi: 10.1371/journal.pbio.3002091 (PMC10187937; doi:10.1371/journal.pbio.3002091)
Supplement: S2 Fig — (PDF) [file pbio.3002091.s015.pdf]

S2 Fig. Elemental analysis results of isolated streptothricin D from commercially available nourseothricin sulfate. The percent compositions align with a molecular formula of $C_{31}H_{58}N_{12}O_{10}$ • 5/2 H_2SO_4 • 5 H_2O.

C	H	N	O
31.41 %	5.49 %	15.00 %	35.78 %

S
7.06 %
