# Supplementary material for: Streptothricin F is a bactericidal antibiotic effective against highly drug-resistant gram-negative bacteria that interacts with the 30S subunit of the 70S ribosome
Source: PLoS Biol. 2023 May 16;21(5):e3002091. doi: 10.1371/journal.pbio.3002091 (PMC10187937; doi:10.1371/journal.pbio.3002091)
Supplement: S3 Fig — The numbering scheme for streptothricin F and streptothricin D. In the 1H NMR, distinct chemical shifts for protons attached to the same carbon atom are labeled Ha and Hb, respectively. (PDF) [file pbio.3002091.s016.pdf]

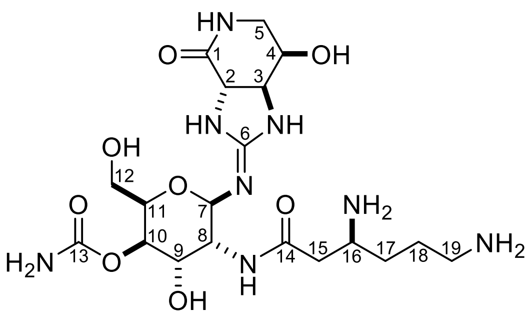

Streptothricin F

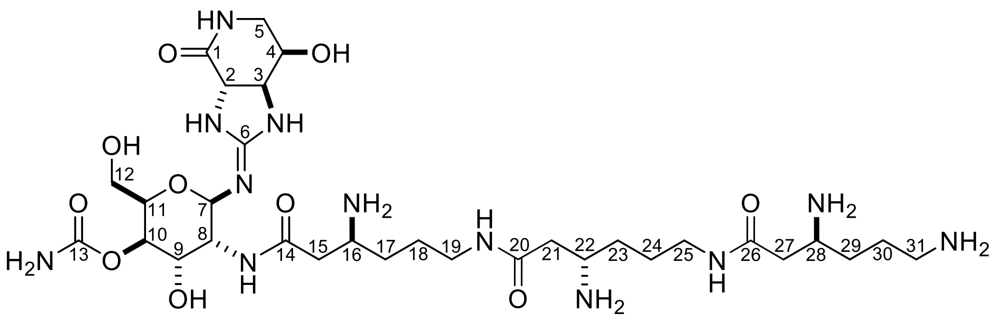

Streptothricin D

S3 Fig. Assignment of Chemical Shifts. The numbering scheme for Streptothricin F and Streptothricin D. In the ^{1}H NMR, distinct chemical shifts for protons attached to the same carbon atom are labeled H_a and H_b, respectively.
