# Supplementary material for: Streptothricin F is a bactericidal antibiotic effective against highly drug-resistant gram-negative bacteria that interacts with the 30S subunit of the 70S ribosome
Source: PLoS Biol. 2023 May 16;21(5):e3002091. doi: 10.1371/journal.pbio.3002091 (PMC10187937; doi:10.1371/journal.pbio.3002091)
Supplement: S4 Fig — Nourseothricin MIC was 2 μg/mL. (PDF) [file pbio.3002091.s017.pdf]

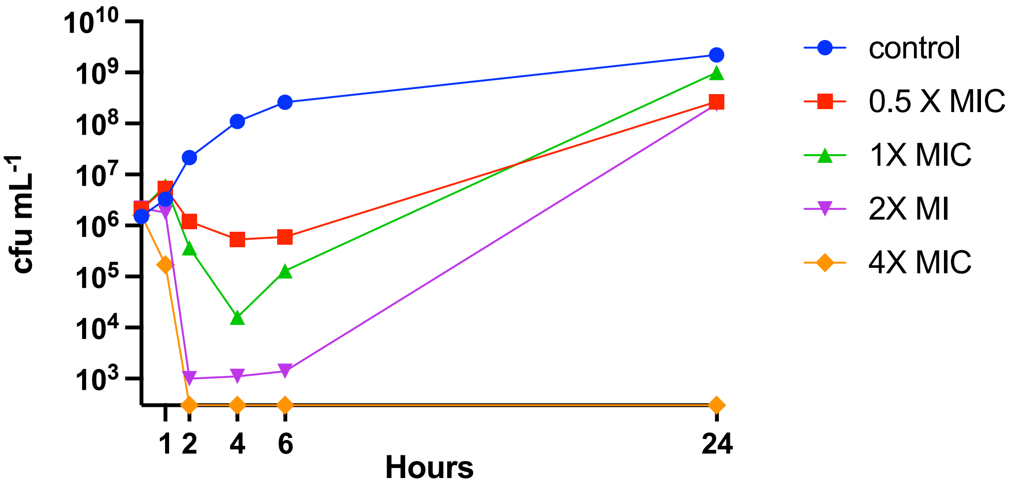


**S4 Fig. Rapid bactericidal activity of nourseothricin against the carbapenem-resistant *Acinetobacter baumannii* isolate, MSRN1450.** Nourseothricin MIC was 2µg/mL. See also S2 Data.
