# Supplementary material for: Streptothricin F is a bactericidal antibiotic effective against highly drug-resistant gram-negative bacteria that interacts with the 30S subunit of the 70S ribosome
Source: PLoS Biol. 2023 May 16;21(5):e3002091. doi: 10.1371/journal.pbio.3002091 (PMC10187937; doi:10.1371/journal.pbio.3002091)
Supplement: S5 Fig — (PDF) [file pbio.3002091.s018.pdf]

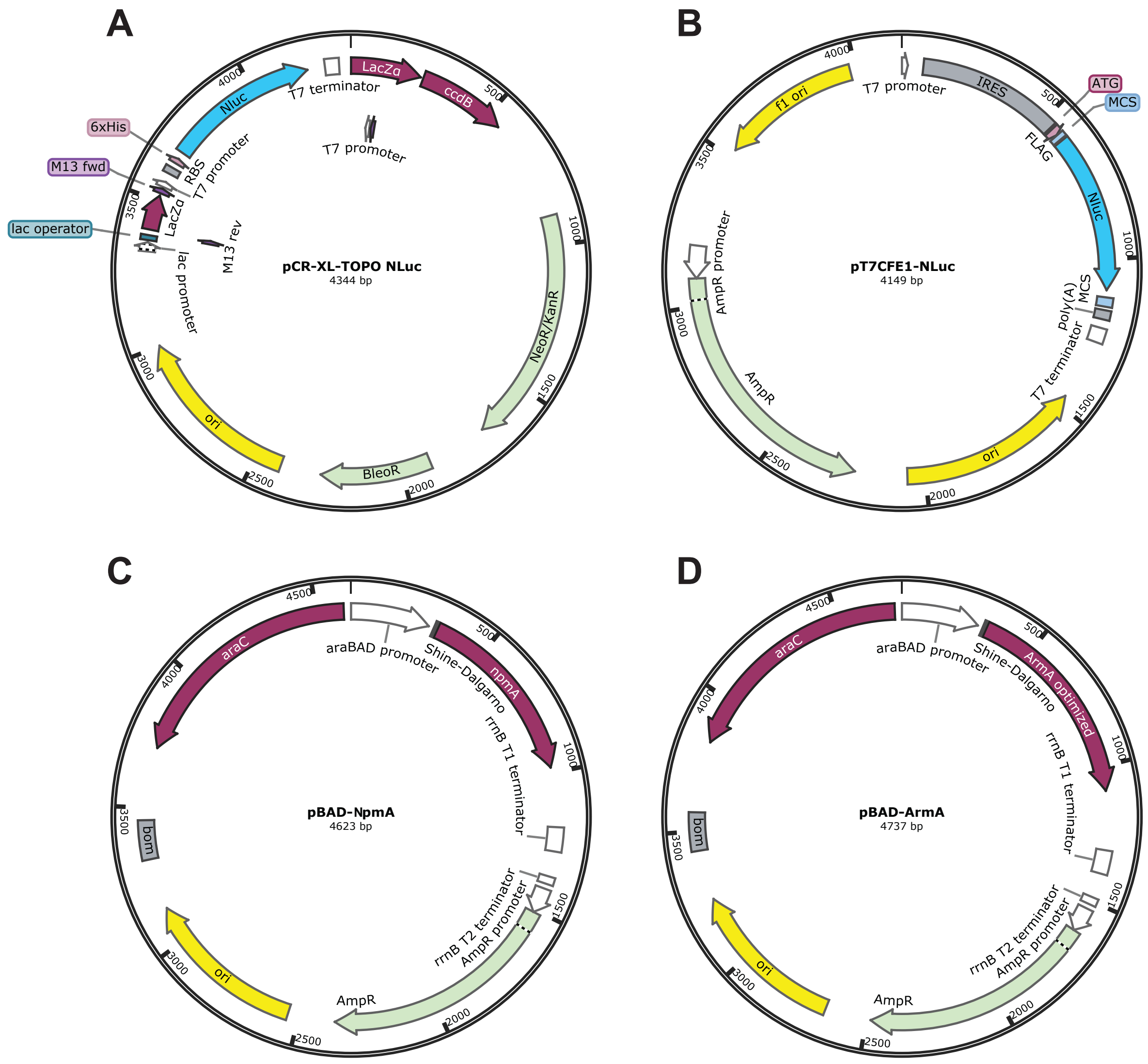

S5 Fig. Maps of vectors used in *in vitro* translational and ribosomal methylase inhibition analysis. (A) pCR-XL-TOPO NLuc contains the nanoluciferase gene (NLuc) located downstream of both lac and T7 polymerase promoters and was used for analysis of inhibition of prokaryotic translation by S-F, S-D and nourseothricin. **(B)** pT7CEF1-NLuc contains the NLuc gene located downstream of a T7 polymerase promoter and IRES sequence and was used for analysis of inhibition of eukaryotic translation by streptothricins. **(C)** pBAD-NpmA contains a codon-optimized npmA gene (S17 Fig) that encodes a 16S rRNA A1408 methylase that blocks activity of all known 2-deoxystreptamine-based aminoglycosides including apramycin. **(D)** pBAD-ArmA contains a codon-optimized armA gene (S17 Fig) that encodes a 16S rRNA G1405 methylase that blocks activity of 4,6-disubstituted deoxystreptamine (DOS) aminoglycosides such as gentamicin and does not block the activity of apramycin. Constructs were confirmed by Sanger sequencing.
