# Supplementary material for: Streptothricin F is a bactericidal antibiotic effective against highly drug-resistant gram-negative bacteria that interacts with the 30S subunit of the 70S ribosome
Source: PLoS Biol. 2023 May 16;21(5):e3002091. doi: 10.1371/journal.pbio.3002091 (PMC10187937; doi:10.1371/journal.pbio.3002091)
Supplement: S6 Fig — (PDF) [file pbio.3002091.s019.pdf]

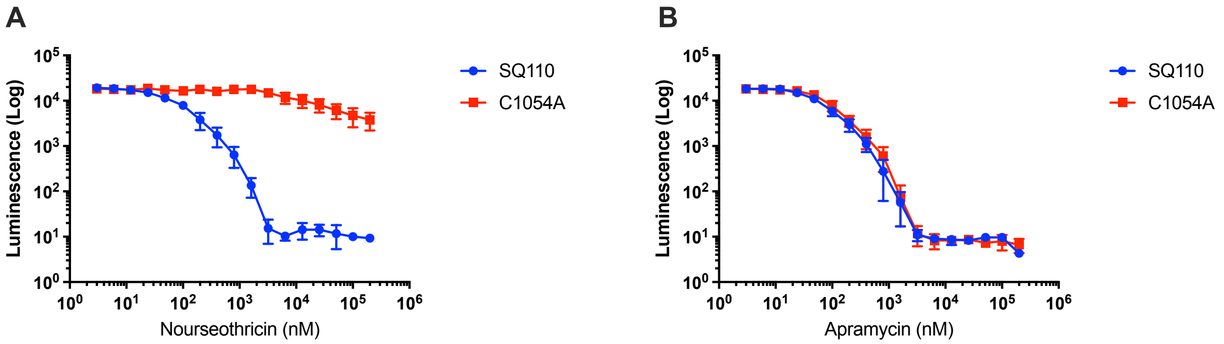

S6 Fig. Resistance of C1054A mutant ribosome to nourseothricin in *in vitro* translation assays. *In vitro* coupled transcription-translation extracts were made from parent, single ribosomal operon strain, SQ110, and from a spontaneous 16S rRNA C1054A nourseothricin resistance mutant, N1 (see Table 1). In these assays, luminescence provided a readout of translation based upon expression and translation of nanoluciferase gene construct added to the reaction mixtures. **(A)** Nourseothricin inhibited translation of SQ110 parent (blue circles) with an IC_{50} and inhibition curve similar to effects observed with commercial extracts made from *E. coli* containing the normal complement of ribosomal operons (Fig 3). In contrast, translation extracts prepared from the C1054A mutant (red squares) were resistant to inhibition at the highest concentrations tested. **(B** In contrast, *in vitro* translation extracts from both parent SQ110 and C1054A strains were similarly inhibited by the positive control, apramycin, confirming that the resistance to inhibition observed with the C1054A extract was nourseothricin specific. See also S6 Data.
