# Supplementary material for: Streptothricin F is a bactericidal antibiotic effective against highly drug-resistant gram-negative bacteria that interacts with the 30S subunit of the 70S ribosome
Source: PLoS Biol. 2023 May 16;21(5):e3002091. doi: 10.1371/journal.pbio.3002091 (PMC10187937; doi:10.1371/journal.pbio.3002091)
Supplement: S8 Fig — (PDF) [file pbio.3002091.s021.pdf]

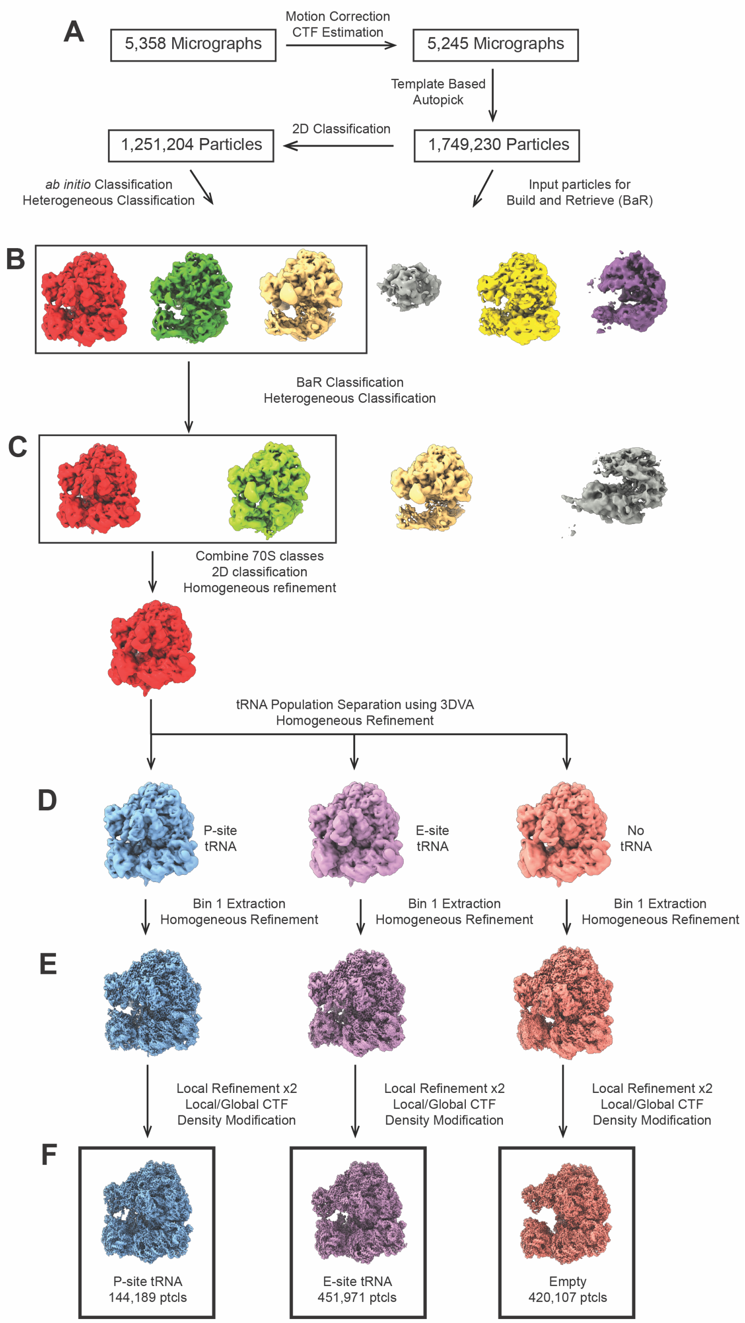

S8 Fig. S-D Cryo-EM processing workflow. (A) 5,358 micrographs were processed and classified to give rise to an initial particle pool of 1,251,204 particles. **(B)** *ab initio* classification gave rise to initial classes. **(C)** A modified build-and-retrieve (BaR) approach was used to generate the full particle set. Complete 70S classes were combined and refined. **(D)** Full particle set was separated based off of tRNA population using 3D variability analysis in cryoSPARC. **(E)** Particles were extracted at full resolution and refined with homogeneous refinement. (F) Final maps refined with local/global CTF correction and focused local refinements and composite maps generated using Relion postprocessing.
