# Supplementary material for: Streptothricin F is a bactericidal antibiotic effective against highly drug-resistant gram-negative bacteria that interacts with the 30S subunit of the 70S ribosome
Source: PLoS Biol. 2023 May 16;21(5):e3002091. doi: 10.1371/journal.pbio.3002091 (PMC10187937; doi:10.1371/journal.pbio.3002091)
Supplement: S10 Fig — (PDF) [file pbio.3002091.s023.pdf]

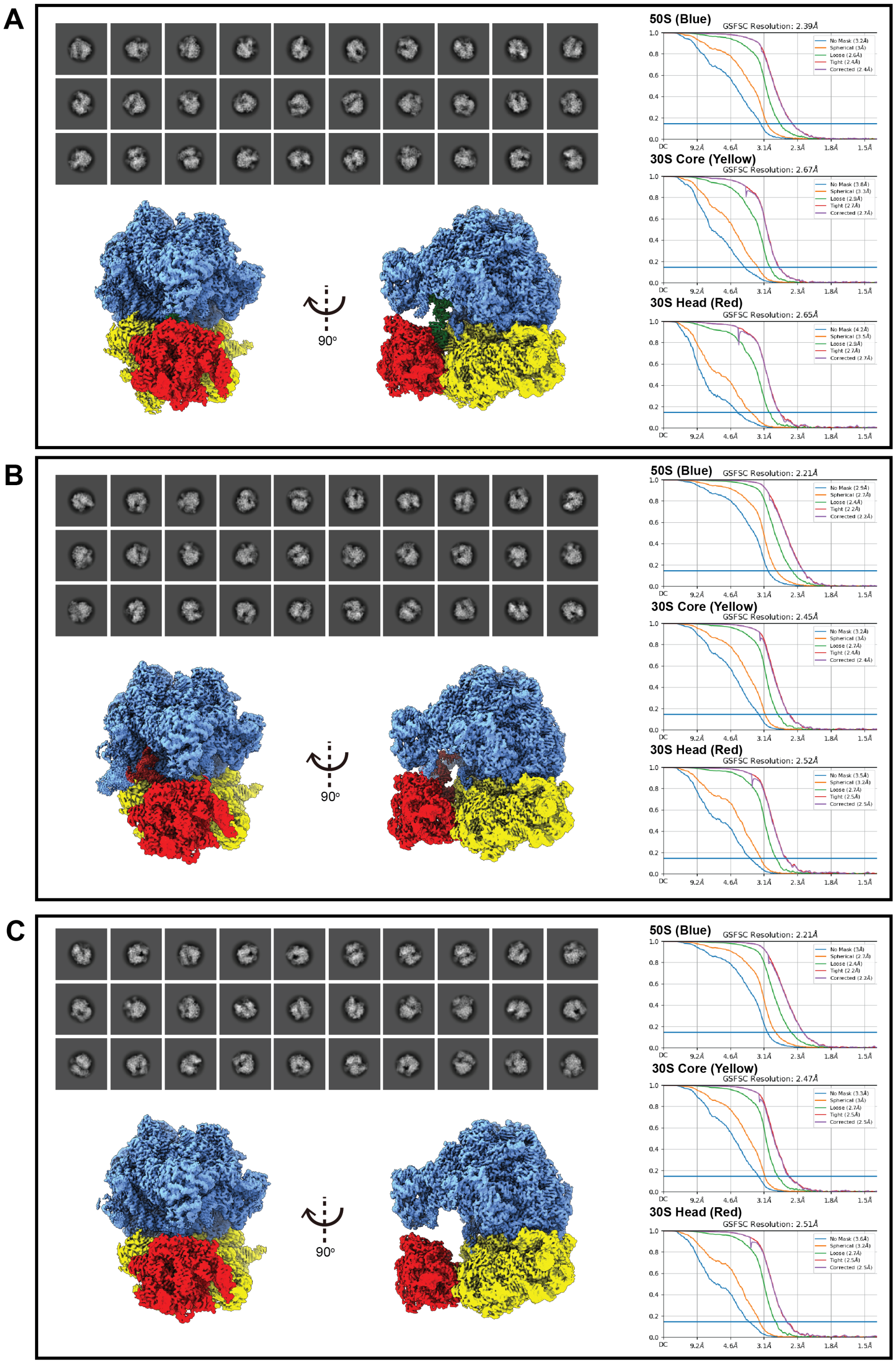

S10 Fig. Final refined maps of *A. baumannii* 70S – S-D complexes. (A) 2D classes (top left), Final map (bottom left) and GS-FSC curves of focus-refined regions of the P-site tRNA 70S – S-D complex. **(B)** 2D classes (top left), Final map (bottom left) and GS-FSC curves of focus-refined regions of the E-site tRNA 70S – S-D complex. **(C)** 2D classes (top left), Final map (bottom left) and GS-FSC curves of focus-refined regions of the empty 70S – S-D complex. In all figures, maps are color coded by focused refinement (50S = blue, 30S core = yellow and 30S head = red). See also S8 Data.
