# Supplementary material for: Streptothricin F is a bactericidal antibiotic effective against highly drug-resistant gram-negative bacteria that interacts with the 30S subunit of the 70S ribosome
Source: PLoS Biol. 2023 May 16;21(5):e3002091. doi: 10.1371/journal.pbio.3002091 (PMC10187937; doi:10.1371/journal.pbio.3002091)
Supplement: S11 Fig — (PDF) [file pbio.3002091.s024.pdf]

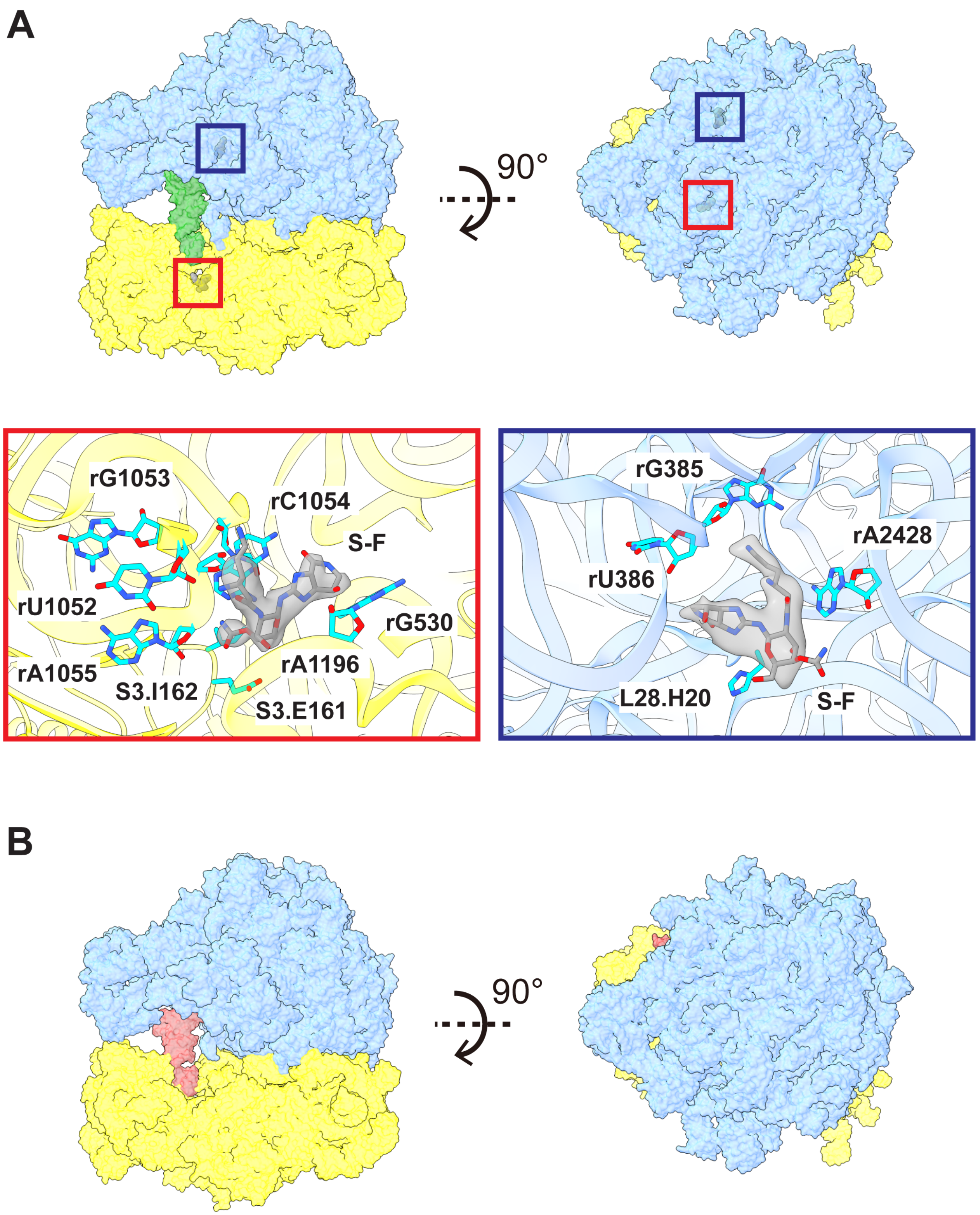

S11 Fig. S-F binding sites in the *A. baumannii* 70S ribosome **(continued on next page).**

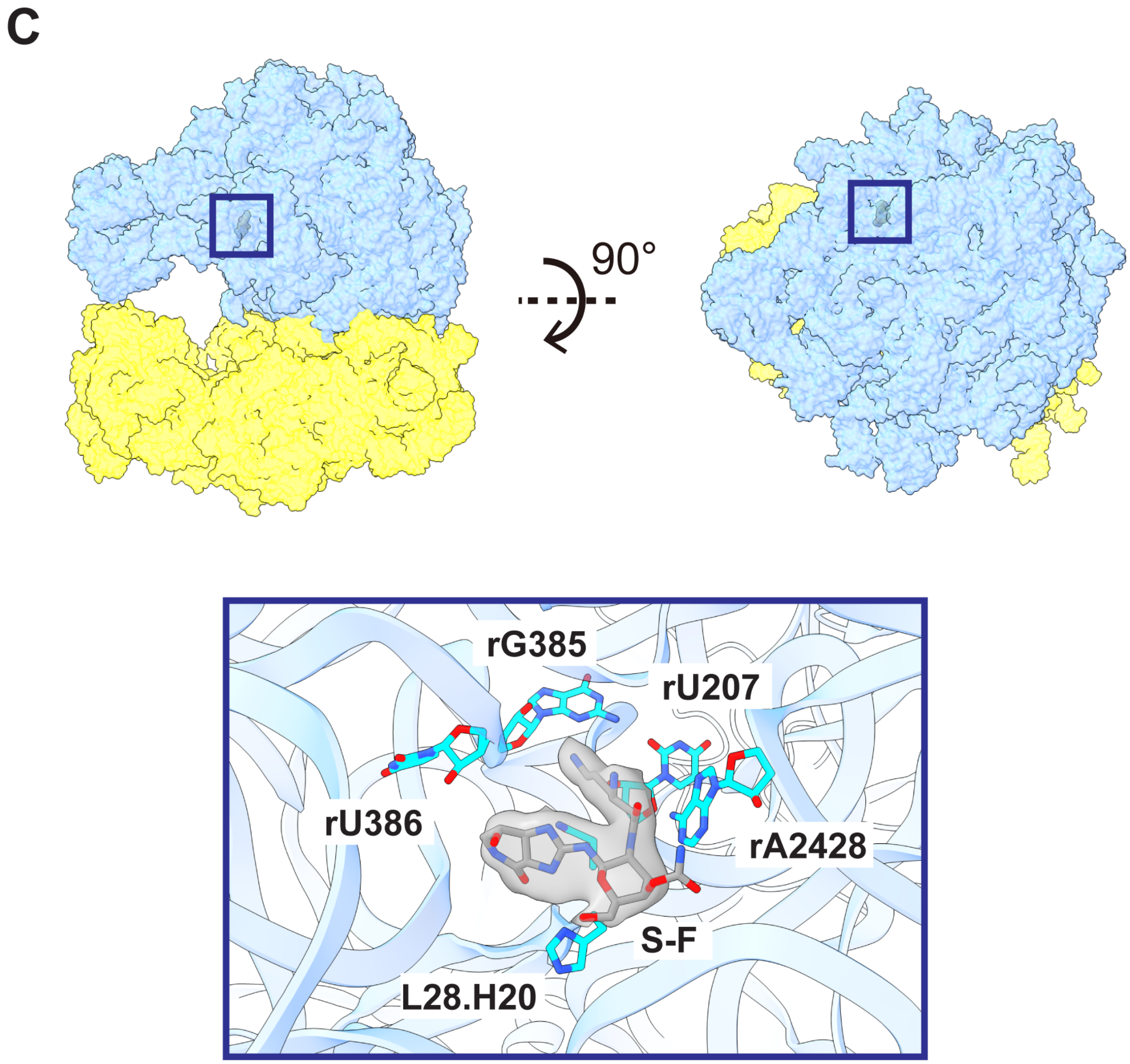

S11 Fig. S-F binding sites in the *A. baumannii* 70S ribosome. (A) Two binding sites were found in the P-site 70S: one in the 30S (red, site 1) and another in the 50S (blue, site 2). **(B)** In the 70S with E-site tRNA, no S-F sites were found. This is likely due to rotation of the 30S head and occlusion of the 50S site by the de-acylated end of the tRNA. **(C)** In the empty 70S, bound S-F was only detected in the 50S site (blue, site 2). 16S rRNA, *E. coli* numbering.
