# Supplementary material for: Streptothricin F is a bactericidal antibiotic effective against highly drug-resistant gram-negative bacteria that interacts with the 30S subunit of the 70S ribosome
Source: PLoS Biol. 2023 May 16;21(5):e3002091. doi: 10.1371/journal.pbio.3002091 (PMC10187937; doi:10.1371/journal.pbio.3002091)
Supplement: S12 Fig — (PDF) [file pbio.3002091.s025.pdf]

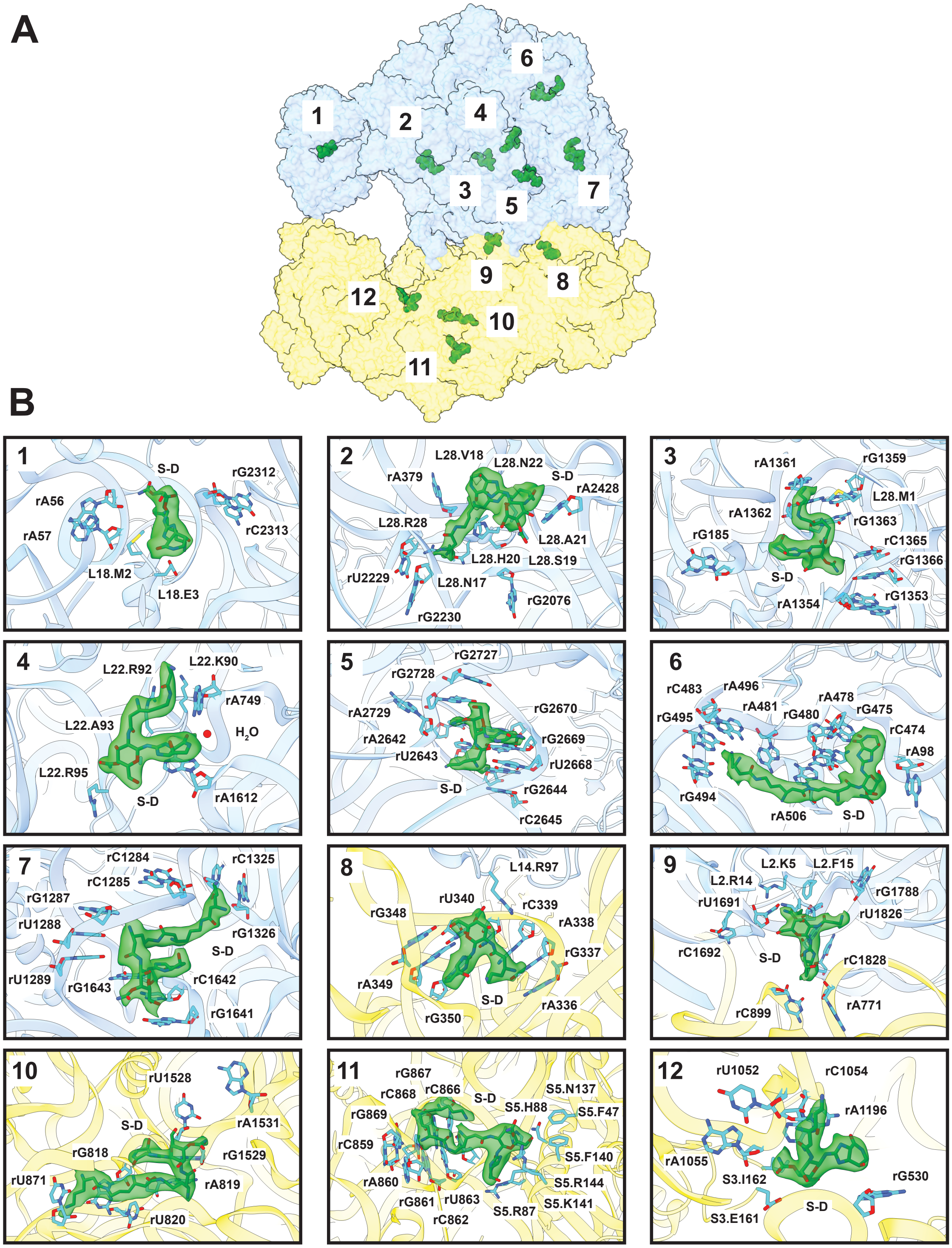

S12 Fig. S-D binding sites in the *A. baumannii* 70S ribosome. (A) Location of the 12 S-D binding sites located in the 70S ribosome. **(B)** Zoomed in views of the 12 binding sites in the ribosome. Only number 12 is perfectly conserved between S-F and S-D. Binding at all sites was found for empty, P-site and E-site 70S complexes, with the exception of site #2 in E-site 70S, which overlaps with the deacylated end of the E-site tRNA and therefore is unavailable for binding. 16S rRNA labeled with *E. coli* numbering.
