# Supplementary material for: Streptothricin F is a bactericidal antibiotic effective against highly drug-resistant gram-negative bacteria that interacts with the 30S subunit of the 70S ribosome
Source: PLoS Biol. 2023 May 16;21(5):e3002091. doi: 10.1371/journal.pbio.3002091 (PMC10187937; doi:10.1371/journal.pbio.3002091)
Supplement: S13 Fig — (PDF) [file pbio.3002091.s026.pdf]

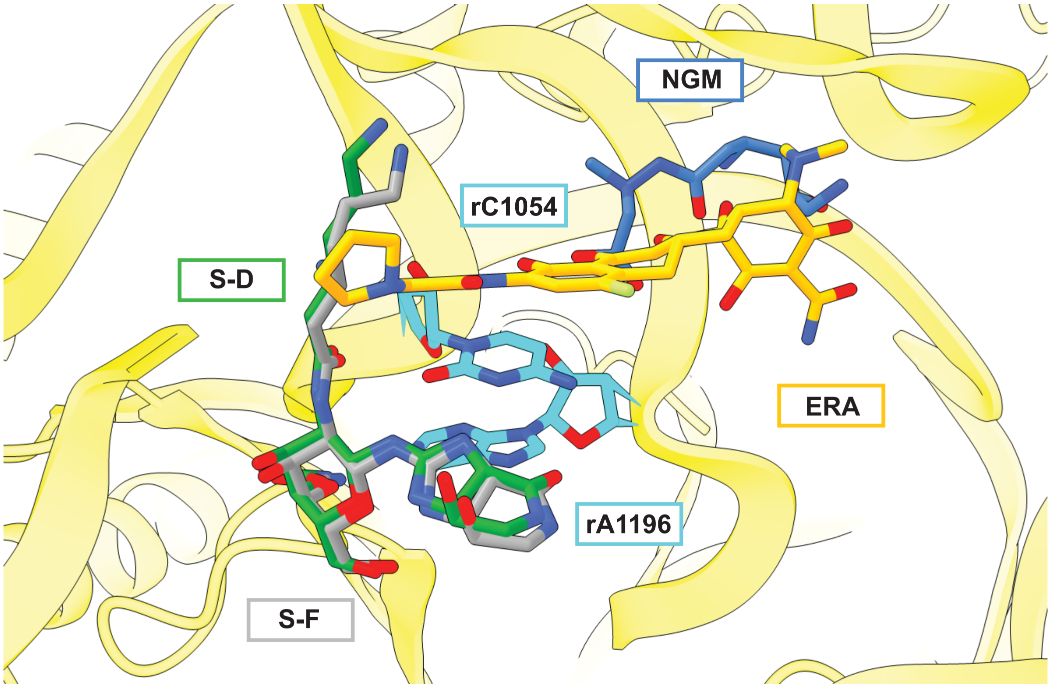

S13 Fig. Similarities in the streptothricin binding site. Comparisons between S-F (gray), S-D (green), eravacycline (ERA, Orange, 7M4X) and negamycin (NGM, Purple, 4WF1) show all three occupy a similar area with distinct stabilizing interactions near C1054 (*E. coli* numbering).
