# Supplementary material for: Streptothricin F is a bactericidal antibiotic effective against highly drug-resistant gram-negative bacteria that interacts with the 30S subunit of the 70S ribosome
Source: PLoS Biol. 2023 May 16;21(5):e3002091. doi: 10.1371/journal.pbio.3002091 (PMC10187937; doi:10.1371/journal.pbio.3002091)
Supplement: S14 Fig — (PDF) [file pbio.3002091.s027.pdf]

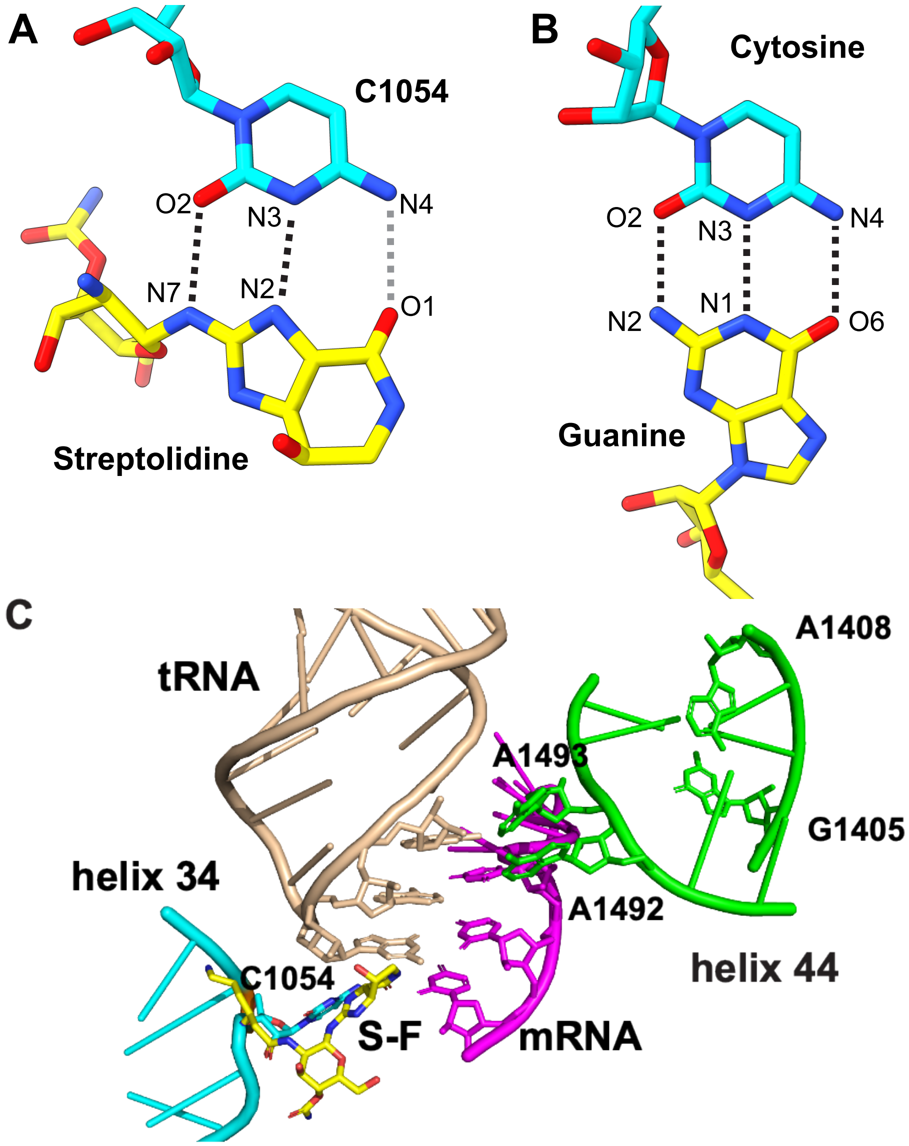

S14 Fig. Proposed S-F mechanism of action. (A) S-F streptolidine binding to C-1054 (*E. coli* numbering) resembles **(B)** G:C Watson-Crick base pairing. Hydrogen bonds (black dashes); electrostatic interaction (grey dashes). **(C)** This binding places the S-F streptolidine moiety in contact with the wobble base in the anti-codon stem loop (ASL). Shown is an overlay of S-F with the A-site ASL, mRNA and helix 44 from PDB 4V8B, based on alignment of C1054 from our S-F structure with C1054 of 4V8B. Interaction of S-F with the ASL wobble base is proposed to stabilize non-cognate tRNA binding to the A-site codon and lead to miscoding. This idea is analogous to the stabilization of A1492 and A1493 in the flipped-out position in response to aminoglycosides, such as paromomycin in PDB 1IBM, thus leading to the inappropriate stabilization of non-cognate tRNA binding to the A-site. Here the flipped out A1492 and A1493 are shown probing the minor groove of the first two base pairs of the A-decoding site (codon numbering), while streptolidine interacts with the third.
