# Supplementary material for: Streptothricin F is a bactericidal antibiotic effective against highly drug-resistant gram-negative bacteria that interacts with the 30S subunit of the 70S ribosome
Source: PLoS Biol. 2023 May 16;21(5):e3002091. doi: 10.1371/journal.pbio.3002091 (PMC10187937; doi:10.1371/journal.pbio.3002091)
Supplement: S15 Fig — (PDF) [file pbio.3002091.s028.pdf]

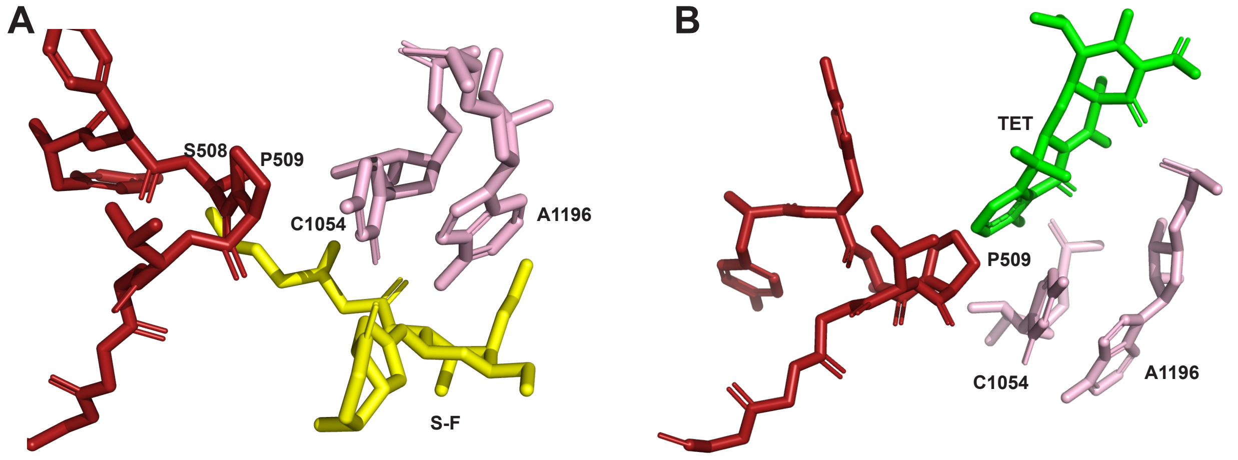

S15 Fig. TetM does not clash with S-F. **(A)** C1054 from our S-F structure and PDB 3J9Y were aligned to assess the overlap of TetM with S-F: TetM, portion of loop 3 of domain IV (red); S-F (yellow); 16S rRNA C1054 and A1196 (yellow). The streptolidine of S-F which forms the major contact with C1054 interacts with the edge of the cytosine pyrimidine ring, away from TetM P509, which makes stacking interactions with C1054. Although, the terminal 6-amino group of the S-F β-lysine clashes with TetM S508, the β-lysine hexanoic amino acid can likely tolerate multiple rotameric states that avoid this clash. **(B)** In contrast, alignment of PDB 3J9Y with the *E. coli* ribosome bound to tetracycline in PDB 5J5B shows steric clash between P509 and the tetracycline D-ring (TET, green) consistent with TetM's ability to block access of tetracycline to its helix 34 binding site.
