# Supplementary material for: Streptothricin F is a bactericidal antibiotic effective against highly drug-resistant gram-negative bacteria that interacts with the 30S subunit of the 70S ribosome
Source: PLoS Biol. 2023 May 16;21(5):e3002091. doi: 10.1371/journal.pbio.3002091 (PMC10187937; doi:10.1371/journal.pbio.3002091)
Supplement: S16 Fig — (PDF) [file pbio.3002091.s029.pdf]

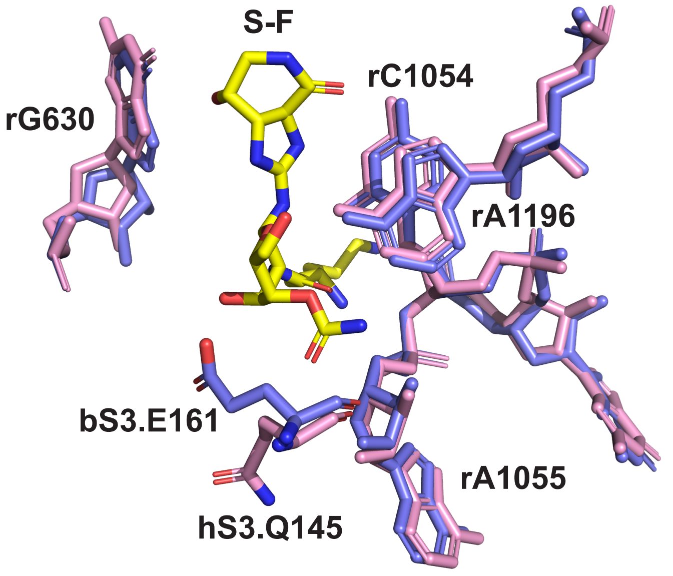

S16 Fig. Structural conservation of the S-F binding site in the human small ribosome subunit. The structural basis for the relatively selective inhibition of prokaryotic translation by streptothricins is not clear as the binding pocket of S-F bound by helix 34, helix 18 (bacterial rG630), and rProtein s3 (bacterial s3.E161) is conserved in the human ribosome (7TQL). S-F (yellow), *A. baumannii* (purple), and human (pink).16S rRNA nucleotides shown with *E. coli* numbering. The carbonyl group from the C-terminus of respective s3 rProtein amino acids from *A. baumannii* (bS3.E161) and human (hs3.Q145) are similarly positioned to make a predicted polar contact with the S-F carbamoyl moiety.
