# Supplementary material for: Streptothricin F is a bactericidal antibiotic effective against highly drug-resistant gram-negative bacteria that interacts with the 30S subunit of the 70S ribosome
Source: PLoS Biol. 2023 May 16;21(5):e3002091. doi: 10.1371/journal.pbio.3002091 (PMC10187937; doi:10.1371/journal.pbio.3002091)
Supplement: S17 Fig — (PDF) [file pbio.3002091.s030.pdf]

S17 Fig. Codon-optimized 16S rRNA methyltransferase resistance gene sequence.

armA
GCTAAGGAGGTAACATATGGACAAGAATGACGTGGTCAAAAAAATTCTGGAAAGCA
AAAAATACGAAAATTTGGATTCCGACATTGTCGAGAAAGTAGTATCCATTTCAGAG
AAGAAATATAAGCTTAAAGAAGTGGAAAATTACAGCAAAAAGAAACTTCATCAAAT
TTGGGGAAGTTACTACTCTGCGTATCCTAACTGGGATAAACTTTTAAAGAAGTACAA
CCAAGGCCAATTATCTATTGAGGATCTGCTTAAAATTCACAGCTCCACGAATGAACG
TGTCGCGACACTTAATGACTTCTACACTTATGTATTTGGTAATATCAAACATGTCTCG
TCTATCCTTGACTTCGGTTGCGGCTTCAACCCGTTGGCATTGTATCAGTGGAATGAA
AATGAAAAGATCATTTACCATGCTTACGATATCGACCGTGCAGAGATTGCTTTCTTA
AGCAGTATCATCGGCAAATTAAAAACGACGATTAAGTACCGCTTTTTGAATAAGGA
AAGTGACGTCTATAAGGGGACATATGACGTAGTATTTCTGTTGAAGATGTTGCCTGT
CCTTAAACAACAAGACGTCAATATTTTAGACTTTCTGCAATTATTCCATACACAGAA
CTTCGTAATTTCCTTCCCTATCAAAAGCCTGTCTGGAAAGGAAAAAGGCATGGAGGA
AAATTACCAGTTGTGGTTCGAATCTTTCACCAAAGGTTGGATTAAAATTTTGGACTC
GAAAGTGATTGGTAACGAATTGGTATACATCACTTCGGGCTTCCAAAAGTAA

npmA
GCTAAGGAGGtTAACATATGCTGATTCTGAAAGGAACTAAAACGGTAGATTTAAGTA
AAGACGAACTTACTGAAATTATCGGCCAATTTGATCGCGTTCACATTGATTTGGGCA
CAGGAGATGGCCGCAACATCTATAAGCTGGCGATTAATGATCAGAATACTTTTTATA
TCGGCATCGACCCAGTTAAGGAGAATCTGTTTGATATCTCAAAGAAAATTATTAAGA
AGCCTTCCAAGGGGGGACTTAGTAATGTAGTGTTCGTGATCGCCGCTGCGGAATCTT
TGCCCTTTGAGCTTAAAAACATTGCCGACTCCATCTCGATTCTTTTTCCATGGGGCAC
ACTGTTGGAGTATGTAATCAAACCCAACCGCGACATCTTATCAAATGTAGCAGACCT
TGCTAAAAAAGAGGCTCACTTTGAGTTTGTGACGACATATAGCGATAGTTACGAAG
AAGCTGAAATCAAGAAGCGTGGCCTGCCACTTCTTAGCAAGGCTTACTTTTTGTCTG
AACAATATAAGGCTGAACTTTCTAATAGTGGATTTCGTATCGACGATGTGAAGGAAC
TTGACAATGAGTATGTCAAGCAATTTAATAGCCTGTGGGCAAAGCGTTTAGCATTCG
GTCGTAAGCGCTCGTTCTTCCGCGTGTCCGGTCATGTATCGAAACACTAA
